# mTORC2 Phosphorylation of GSDME‐N Drives Cullin4B‐Mediated Proteasomal Degradation to Suppress Pyroptosis and Confer Radioresistance in Small Cell Lung Cancer

**DOI:** 10.1002/advs.75844

**Published:** 2026-05-27

**Authors:** Qing‐qing Xu, Ci‐ming Sun, Sui‐xian Zhang, Rui Li, Chen‐fei Wu, Zai‐shan Lin, Li Li, Run‐zhe Chen, Qi‐wen Li, Yuan‐yuan Chen, Xuan Li, Ming Chen

**Affiliations:** ^1^ State Key Laboratory of Oncology in South China Guangdong Key Laboratory of Nasopharyngeal Carcinoma Diagnosis and Therapy Guangdong Provincial Clinical Research Center for Cancer Sun Yat‐sen University Cancer Center Guangzhou Guangdong P. R. China; ^2^ Department of Radiation Oncology Sun Yat‐sen University Cancer Center Guangzhou Guangdong P. R. China; ^3^ United Laboratory of Frontier Radiotherapy Technology of Sun Yat‐Sen University & Chinese Academy of Sciences Ion Medical Technology Co Guangzhou Guangdong P. R. China; ^4^ VIP Region Sun Yat‐sen University Cancer Center Guangzhou P. R. China

**Keywords:** GSDME, mTORC2, pyroptosis, radioresistance, small cell lung cancer

## Abstract

Radioresistance is a main reason for treatment failure in patients with small cell lung cancer (SCLC). Consequently, it is important to determine the key mechanism and explore effective strategies to prevent SCLC radioresistance. We use an unbiased CRISPR screen to identify GSDME, a member of the Gasdermin (GSDM) family, as a critical driver of radiosensitivity in SCLC. Furthermore, we identify mTORC2 facilitates SCLC radioresistance by inhibiting GSDME‐N‐mediated pyroptosis. Mechanistically, mTORC2 phosphorylates GSDME‐N at serine 114 (S114), promoting the recruitment of the CUL4B‐RBBP4 E3 ubiquitin ligase complex. This complex mediates K48‐linked ubiquitination of GSDME‐N at lysine 41 (K41), leading to its proteasomal degradation. Clinically, elevated mTORC2 is linked to an unfavorable prognosis in SCLC patients. The study reveals mTORC2 phosphorylates GSDME‐N and promotes its Cullin4B‐mediated proteasomal degradation to suppress pyroptosis and drive radioresistance in SCLC.

## Graphical abstract

Radioresistance severely limits the efficacy of therapies for small cell lung cancer (SCLC). This study reveals a novel mechanism of resistance driven by the active suppression of pyroptosis. Specifically, the mTORC2 complex directly phosphorylates GSDME‐N and promotes its CUL4B‐mediated ubiquitination and proteasomal degradation. Targeting mTORC2‐CUL4B‐GSDME axis offers a potent strategy to restore SCLC radiosensitivity.

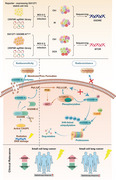

## 1 Introduction

Small cell lung cancer (SCLC) is a distinct pathological subtype of lung cancer, accounting for approximately 15% of all cases [1, 2]. It is characterized by poor differentiation and high malignancy, with concurrent chemoradiotherapy being the mainstay of treatment [3]. Although SCLC initially responds well to radiotherapy, it frequently acquires therapeutic resistance, leading to recurrence and metastasis [4]. Identifying the key molecular drivers of radioresistance and developing effective intervention strategies are critical for improving patient survival [5].

Pyroptosis is a form of programmed cell death triggered by the formation of gasdermin(GSDM)‐mediated pores in the plasma membrane [6, 7]. GSDM family proteins possess a C‐terminal autoinhibitory domain and can be cleaved by various upstream caspases, leading to the release of their C‐terminal inhibition. The liberated N‐terminal domain can self‐assemble into an 18‐subunit superstructure that inserts into the plasma membrane to form pores, forcing the release of cellular contents and ultimately resulting in lytic cell death [8, 9]. Pyroptosis has been increasingly recognized as a crucial mechanism in tumor suppression and holds significant clinical potential [10, 11]. However, many cancer cells exhibit low expression of GSDM proteins, limiting their ability to undergo pyroptosis [12]. GSDME is a GSDM subtype gene primarily expressed in epithelial cells, and its low expression has been extensively studied in relation to tumorigenesis and chemotherapy resistance. Notably, enforced expression of GSDME has been shown to restore pyroptotic potential and overcome chemoresistance in tumor cells [13, 14]. Regarding the regulation of GSDME expression, in addition to transcriptional repression due to DNA methylation changes in its promoter region, studies have also reported the degradation of GSDME in tumor tissues. However, the free N‐terminal isoform is the critical determinant for pyroptosis occurrence. Research on the stability of the N‐terminal isoform remains scarce, and the role of GSDME‐mediated pyroptosis in radioresistance in SCLC is still unclear.

Although pyroptosis is profoundly suppressed in radioresistant SCLC, the precise molecular mechanisms driving this phenotype remain elusive. To address this, we established an inducible pyroptosis system and performed an unbiased genome‐wide CRISPR screen, which identified the mammalian target of rapamycin (mTOR) as a critical modulator of pyroptosis in SCLC. mTOR functions within two distinct complexes, mTORC1 and mTORC2, to regulate tumor growth, cell survival, and therapeutic resistance [15, 16, 17]. Guided by the screening result, we investigated how mTOR signaling impinges on pyroptosis. Notably, by performing immunoprecipitation coupled with mass spectrometry targeting GSDME‐N, we identified Cullin 4B (CUL4B), a scaffold protein of the CRL4B E3 ubiquitin ligase complex, as a novel interacting partner [18]. The ubiquitin‐proteasome system (UPS) is the primary pathway for intracellular protein degradation, relying on E3 ligases such as CUL4B to confer substrate specificity [19, 20, 21]. We found that CUL4B directly binds to and ubiquitinates GSDME‐N, leading to its proteasomal degradation and thereby blocking pyroptosis. Importantly, this degradation event is responsible for SCLC resistance. Further mechanistic dissection revealed that mTORC2 directly phosphorylates GSDME‐N, and this phosphorylation promotes the physical association between CUL4B and GSDME‐N, thereby enhancing the ubiquitination and turnover of the pyroptotic effector. Consequently, genetic or pharmacological inhibition of mTORC2 disrupts the phosphorylation of GSDME‐N, reduces its binding to CUL4B, stabilizes GSDME‐N, restores pyroptosis, and resensitizes radioresistant SCLC cells to irradiation. Finally, we validated this mTORC2‐CUL4B‐GSDME mechanism in animal models and clinical SCLC patients, demonstrating its potential as a therapeutic target.

## 2 Results

### 2.1 CRISPR/Cas9 Screen Identifies GSDME‐Mediated Pyroptosis as a Potential Modulator of Radioresistance in SCLC

**FIGURE 1:**
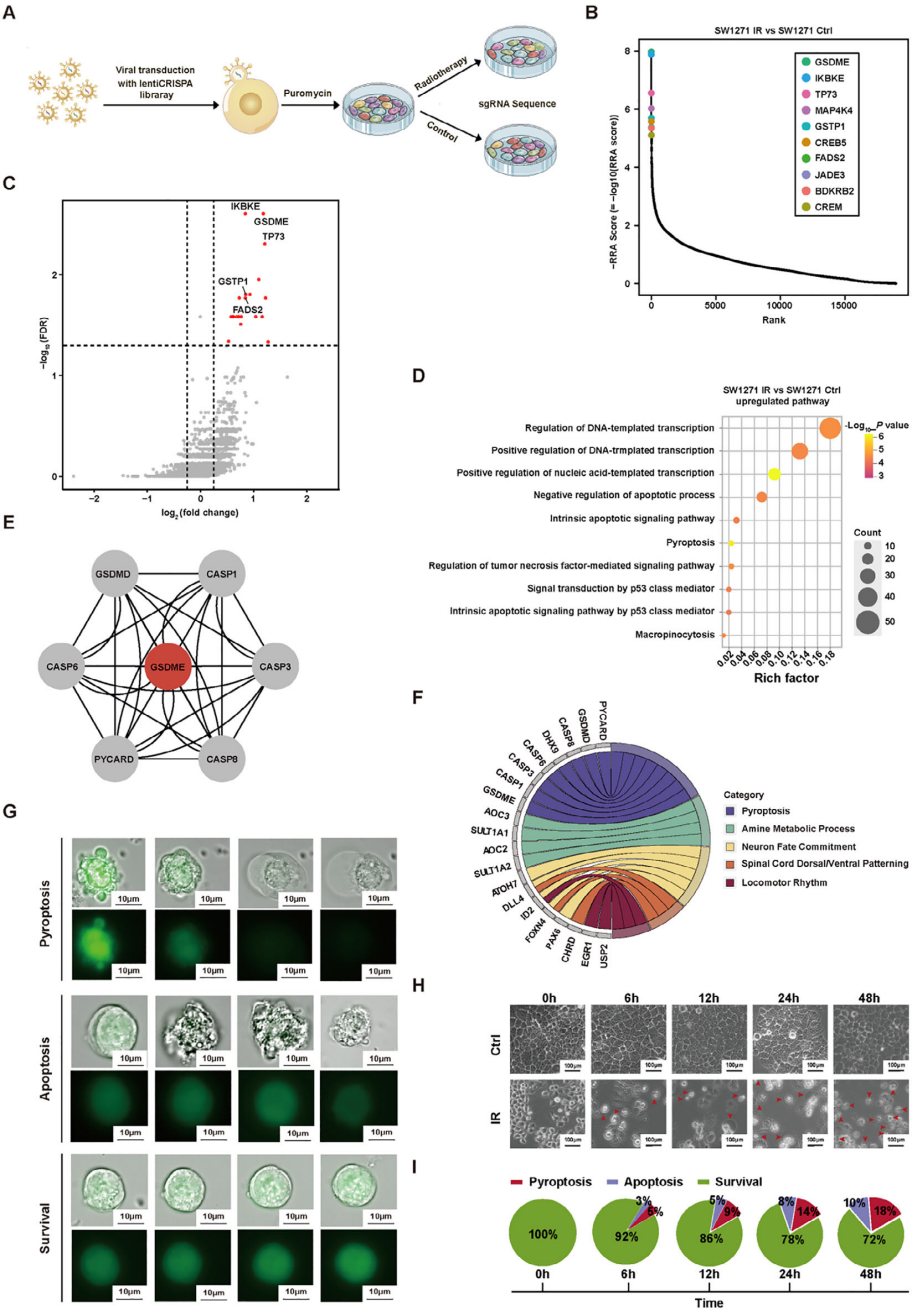
CRISPR/Cas9 screen identifies GSDME‐mediated pyroptosis as a potential modulator of radioresistance in SCLC. (A) Schematic workflow of the genome‐wide CRISPR/Cas9 screening in SW1271 cells treated with or without ionizing radiation (IR, 8 Gy). (B) Top 10 positively selected genes in irradiated SW1271 cells vs. non‐irradiated controls, based on CRISPR screening analysis. (C) Volcano plot illustrates significant candidate genes identified from the comparison of irradiated SW1271 cells vs. non‐irradiated controls. (D) Gene Ontology (GO) enrichment analysis of upregulated pathways in SW1271 cells following IR. The top 10 enriched pathways are shown. (E) Protein interaction network (PPI) network analysis of pyroptosis‐related genes upregulated in radiosensitive vs. radioresistant patients, highlighting central nodes. (F) GO enrichment analysis of RNA‐seq data revealed a positive association between GSDME expression and activation of the pyroptosis pathway. (G) SBC‐2 cells stably expressing GFP were irradiated with 8 Gy and imaged using the CV1000 confocal scanner system showed typical processes of pyroptosis, apoptosis, and survival. (H) Phase‐contrast microscopy showing representative pyroptotic morphology in SBC‐2 cells. (I) Quantification of pyroptotic, apoptotic, and surviving cell populations within 48 h after irradiation (8 Gy). Data are representative of three independent experiments in (G–I).

To uncover genes involved in radioresistance in SCLC, we performed a genome‐wide CRISPR/Cas9 activation screen in SW1271 cells. Cells were either exposed to IR or left untreated and cultured in vitro for 7 days. Following sublethal irradiation, the surviving cells repopulated to reach the target sgRNA library coverage. Subsequently, genomic DNA was extracted, and sgRNA representation was analyzed via deep sequencing (Figure 1A). Using the MAGeCK algorithm, we identified a number of candidate genes enriched in IR‐responsive cells. Notably, GSDME, one of the key genes in pyroptosis ranked among the top 10 (Figure 1B,C). Moreover, GO enrichment analysis revealed significant upregulation of the pyroptosis signaling pathway, including gene were upregulated in the SW1271 IR group (Figure 1D). Meanwhile, in order to verify the results of CRISPR/Cas9 screen, we performed RNA‐seq analysis on tumor tissues from radiosensitive and radioresistant SCLC patients. Bubble plot analysis revealed that the pyroptosis pathway was significantly enriched in radiosensitive samples (Figure S1A), with GSDME playing a central role in this pathway (Figure 1E,F). Subsequently, in order to observe the cell morphology after irradiation, SBC‐2 cells stably expressing GFP were irradiated with 8 Gy and imaged using the CV1000 confocal scanner system, which showed typical processes of pyroptosis, apoptosis, and survival (Figure 1G). To further confirm these observations, SBC‐2 cells were exposed to 8 Gy IR, and the morphology was examined under a phase contrast microscope after 48 h. Notably, dying cells displayed typical features of pyroptosis, including vacuole‐like structures on the plasma membrane and aggregation of cytoplasmic contents at one pole of the cell (Figure 1H). Quantification revealed that 5%–18% of SBC‐2 cells underwent pyroptosis following irradiation (Figure 1I). The hallmark features of pyroptosis include the early formation of multifocal vesicle‐like protrusions on the membrane surface, followed by cytoplasmic swelling with polarized accumulation of cellular contents at one pole of the cell. This culminates in membrane rupture, rapid alteration of membrane permeability, and subsequent leakage of cytoplasmic components. Flow cytometry analysis demonstrated a marked and rapid increase in the proportion of Annexin V/PI double‐positive cells in both SBC‐2 and SW1271 cell lines following 8 Gy irradiation. In contrast to apoptosis, where cells were initially Annexin V‐positive and only later PI‐positive, the simultaneous positivity indicated rapid membrane permeability characteristic of pyroptosis (Figure S1B). Meanwhile, through LDH assays on the supernatants of irradiated SCLC cell lines (SBC‐2 and SW1271), we detected significantly higher LDH activity in the irradiated groups compared to the non‐irradiated controls (Figure S1C). The above results demonstrated that GSDME‐dependent pyroptosis plays a crucial role in SCLC radioresistance.

### 2.2 GSDME‐N‐Mediated Pyroptosis Inhibits Radioresistance in SCLC

**FIGURE 2:**
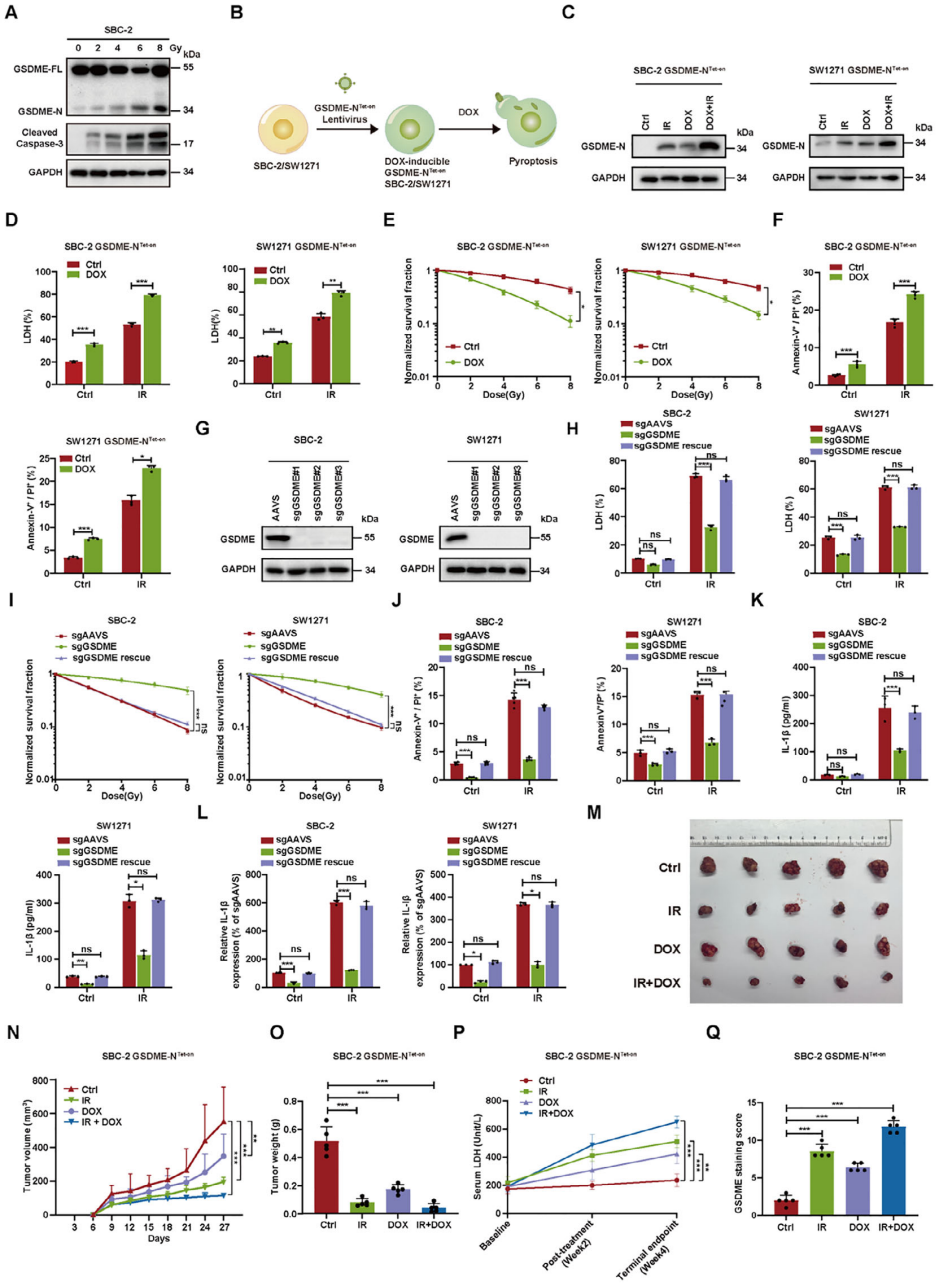
GSDME‐N‐mediated pyroptosis inhibits radioresistance in SCLC. (A) Western blotting analysis of GSDME‐N and Cleaved Caspase‐3 expression in response to increasing IR doses. (B) Schematic of lentiviral transduction strategy used to generate SCLC cells with DOX‐inducible expression of fluorescently tagged GSDME‐N. (C) Western blotting demonstrating the effects of DOX‐inducible expression of GSDME‐N in SBC‐2 and SW1271 GSDME^Tet‐on^ cells following IR. (D–F) LDH release assay, clonogenic survival assay, and Flow cytometry assays showing increased pyroptosis following DOX‐inducible expression of GSDME‐N. (G–I) Western blotting, lactate dehydrogenase (LDH) release assay, and clonogenic survival assay demonstrating the effects of GSDME‐N knockout in SCLC cell lines. (J) Flow cytometric analysis of Annexin V/PI double‐positive cells following GSDME knockdown and restoration in SCLC cell lines. (K) ELISA analysis of IL‐1β in SCLC cells with GSDME knockdown and restoration. (L) Quantitative PCR showing mRNA levels of IL‐1β after GSDME modulation in SCLC cell lines. (M) Representative images of tumors excised at the end of the in vivo experiment. (N, O) Tumor volume and weight of SBC‐2‐GSDME‐N^Tet‐on^ xenografts in nude mice treated with DOX and/or IR (*n* = 5). (P) The release of serum LDH was detected in the nude mice treated with DOX and/or IR (*n* = 5). (Q) Quantification of GSDME expression in xenograft tumor tissues from each treatment group (*n* = 5). Data are representative of three independent experiments in (A, C–L, N–Q). Results are presented as mean ± SD; ^*^ *p* < 0.05, ^**^ *p* < 0.01, ^***^ *p* < 0.001, ns = not significant; *p* values were determined using two‐way ANOVA.

To further investigate the expression level of GSDME in SCLC following irradiation, SBC‐2 cells were subjected to varying doses of irradiation (0, 2, 4, 6, and 8 Gy). Western blotting analysis revealed a dose‐dependent increase in GSDME‐N and Cleaved caspase‐3 expression, this N‐terminal fragment is the active form of GSDME, generated by cleavage of caspase‐3. It mediates membrane permeabilization during pyroptosis, indicating GSDME activation upon irradiation stimulation (Figure 2A and Figure S2A). We performed Western blotting and LDH experiments using pyroptosis agonists and inhibitors. The use of pyroptosis agonist (Cisplatin, DDP) synergized with IR to induce cleavage and activation of GSDME‐N, and increased LDH release. Conversely, pyroptosis inhibitors (Disulfiram) reversed IR‐induced GSDME‐N cleavage and reduced LDH released (Figure S2A,B). In the inactive conformation, GSDME adopts an autoinhibited conformation in which the C‐terminal domain binds to the N‐terminal domain, thereby masking the pore‐forming site and maintaining the protein in a functionally silent state. To specifically investigate the role of pyroptosis in SCLC radiotherapy and to further clarify that its driving mechanism is mediated by GSDME‐N‐dependent membrane perforation, this study employed the Tet‐on inducible gene expression system. Using this system, the GSDME N‐terminal domain was integrated into the genome of SCLC cells via lentiviral vectors, followed by puromycin gradient screening and monoclonal expansion. Subsequently, we successfully established an inducible system in SCLC cells, allowing DOX‐controlled expression of GSDME‐N tagged with GFP at the C‐terminus. This model provides temporal and spatial specificity for studying the regulation of pyroptosis mediated by GSDME‐N (Figure 2B). To validate this model, we treated the GSDME‐N^Tet‐on^ cell line with irradiation, doxorubicin (DOX), or a combination of both. Western blot analysis showed that either treatment alone induced GSDME‐N expression, and their combination resulted in a significant synergistic upregulation (Figure 2C). Further support came from the LDH release assay, colony formation assays and Flow cytometry analysis, which confirmed that the combined treatment increased pyroptosis markers and markedly enhanced radiosensitivity (Figure 2D,F and Figure S2C,D). Since the N‐terminal of GSDME is a post‐translational cleavage product of the protein, it is impossible to specifically knockout it. Therefore, we constructed GSDME knockout cell lines using sgRNA and verified the successful knockout via Western blotting analysis (Figure 2G). Knockout of GSDME significantly reduced radiotherapy‐induced lactate dehydrogenase release, decreased the proportion of Annexin V/PI double‐positive cells, and colony formation assays demonstrated that GSDME deficiency effectively diminished the radiosensitivity of SCLC cells. In addition, EILSA experiments showed decreased secretion of IL‐1β, and IL‐6 following GSDME knockout in irradiated SCLC cells. These findings were further supported by qPCR, which showed reduced expression of inflammatory cytokines. Notably, these effects were reversed upon re‐expression of wild‐type GSDME‐N (Figure 2H,L and Figure S2E–H). In an in vivo xenograft mouse model, DOX administration successfully induced GSDME‐N expression and pyroptosis, while IR was used to mimic therapeutic conditions. Tumor growth (volume and weight) was significantly suppressed in the DOX group, particularly in combination with IR, suggesting a synergistic effect between GSDME‐N‐induced pyroptosis and radiotherapy (Figure 2M–O). Meanwhile, serum LDH release levels of the four groups of mice were detected before treatment, two weeks after treatment, and on the day of execution, and it was found that serum LDH concentration was highest after DOX and IR group (Figure 2P). IHC staining of tumor tissues revealed marked increases in GSDME expression in response to both DOX and IR, further validating pyroptosis activation in vivo (Figure 2Q and Figure S2I). The result suggested that GSDME‐N‐mediated pyroptosis regulates the radiosensitivity of SCLC.

### 2.3 CRISPR Screen Reveals RICTOR/mTORC2 as a Key Regulator of GSDME‐N‐Mediated Pyroptosis

**FIGURE 3:**
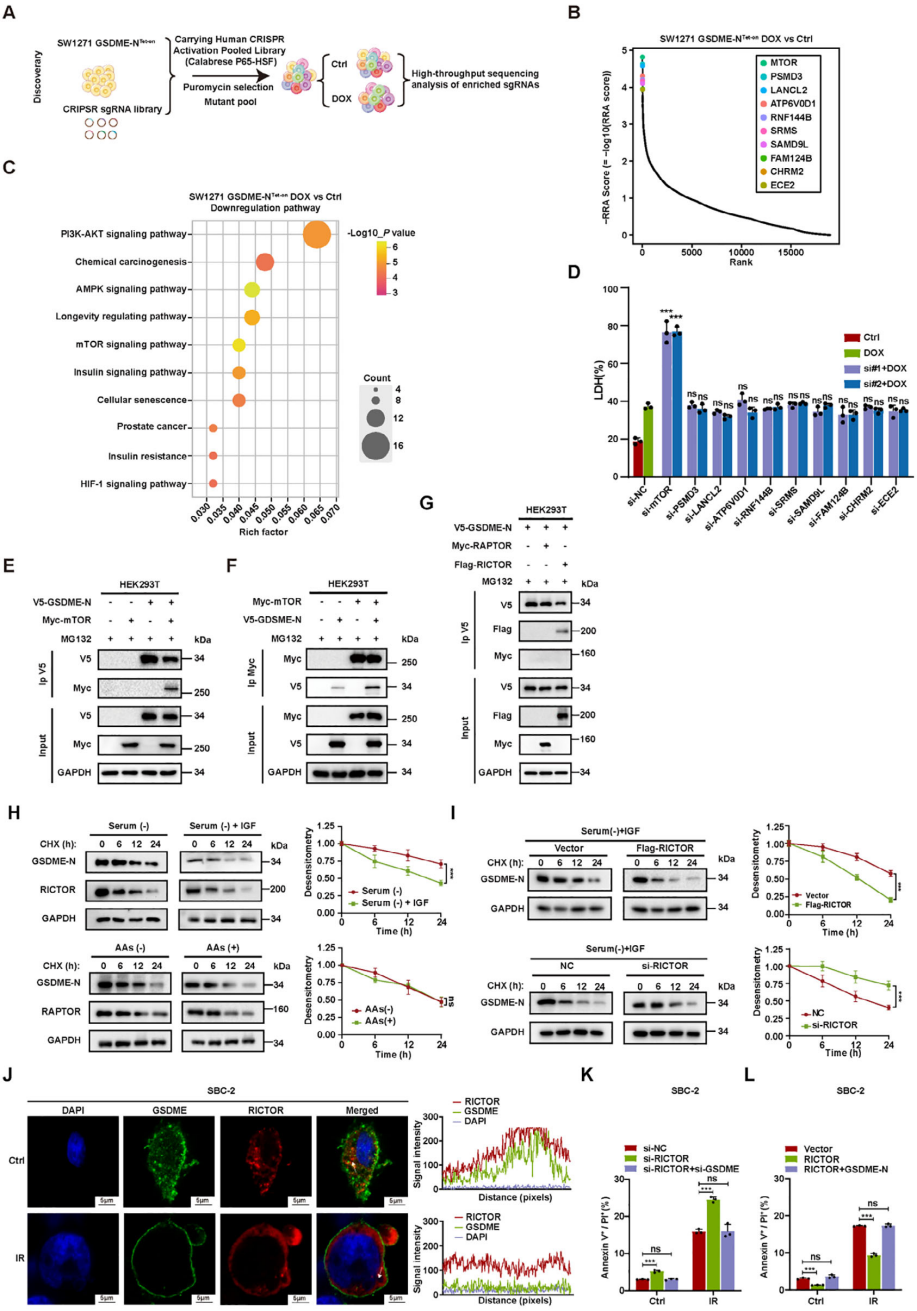
CRISPR screen reveals RICTOR/mTORC2 as a key regulator of GSDME‐N‐mediated pyroptosis. (A) Schematic diagram of the genome‐wide CRISPR‐Cas9 screening workflow in SCLC cells. (B) Scatter plot showing top negatively selected genes in DOX‐treated SW1271 GSDME‐N^Tet‐on^ cells compared with untreated controls. The top 10 genes are labeled. (C) KEGG pathway enrichment analysis of downregulated pathways in irradiated SW1271 GSDME‐N^Tet‐on^ cells. The top 10 pathways are shown. (D) Lactate dehydrogenase (LDH) release assay following siRNA‐mediated knockdown of the top 10 genes in SCLC cells exposed to 8 Gy irradiation. (E, F) Co‐immunoprecipitation (Co‐IP) assays showing the interaction between overexpressed mTOR and GSDME in 293T cells using tag‐specific antibodies. (G) Co‐IP showing selective interaction between GSDME and RICTOR (mTORC2), but not RAPTOR (mTORC1), in SBC‐2 cells. (H) CHX chase assay to assess the stability of GSDME‐N following mTORC1 activation (via amino acid recovery) and mTORC2 activation (via serum starvation and IGF stimulation) in SBC‐2 cells. (I) CHX chase assay showing the effect of RICTOR overexpression or knockdown on GSDME‐N protein stability. (J) Representative confocal immunofluorescence images showing subcellular localization of GSDME‐N and RICTOR in cells with or without IR treatment. Line graphs show the signal intensity of RICTOR, GSDME, and DAPI along the arrow. (K, L) Flow cytometry analysis assessing the impact of knockdown or overexpression of RICTOR on GSDME‐N‐mediated pyroptosis in SCLC cells. Data are representative of three independent experiments in (D–I, J–L). Results are presented as mean ± SD; ^*^ *p* < 0.05, ^**^ *p* < 0.01, ^***^ *p* < 0.001, ns = not significant; *p* values were determined using two‐way ANOVA.

To identify upstream regulators that influence susceptibility to GSDME‐N‐mediated pyroptosis, we conducted a functional screen using a CRISPR activation screen targeting 113 238 sgRNAs in a stable GSDME‐N‐expressing SW1271 cell line established via the Tet‐on system. Under physiological steady‐state conditions, GSDME‐N is inhibited by GSDME‐C and cannot spontaneously trigger pyroptosis; exogenous DOX induction is required to activate the pyroptotic pathway. SW1271 GSDME‐N conditional overexpression cells were subjected to either control or DOX (Figure 3A). In comparison to the pyroptosis‐activated group, the genes more enriched in the control group included mTOR, which ranked particularly high (Figure 3B). Moreover, GO enrichment analysis revealed that the mTOR and PI3K‐AKT signaling pathways were more enriched in the control group compared to the dox‐induced group (Figure 3C). To validate the screen results, we performed an LDH release assay using siRNA targeting the top 10 candidate genes. mTOR knockdown had the most potent effects in enhancing pyroptosis, whereas other genes had minimal influence (Figure 3D). Using irradiation to induce pyroptosis, we next performed parallel qRT‐PCR and western blot analyses after mTOR knockdown or overexpression. These analyses showed that irradiation affected GSDME‐N cleavage levels, and that mTOR modulation altered GSDME‐N protein stability without significantly affecting its mRNA levels (Figure S3A,B). As a widely recognized protein kinase, mTOR can regulate the activity and stability of downstream proteins through direct phosphorylation. Therefore, we speculate that mTOR may phosphorylate GSDME and thereby negatively regulate pyroptosis. CoIP experiments with MYC‐tagged mTOR and V5‐tagged GSDME confirmed their physical interaction (Figure 3E–F). mTOR is a central signaling molecule, forming two complexes: mTORC1, which responds to amino acid levels, and mTORC2, which responds to growth factors. Both complexes play critical roles in tumor initiation, cell cycle regulation, metabolism, and fate determination [22, 23]. To determine which complex primarily interacts with GSDME, we co‐overexpressed GSDME along with either RAPTOR or RICTOR in 293T cells. Co‐IP results showed that RICTOR (mTORC2) interacted with GSDME‐N, while RAPTOR (mTORC1) did not in 293T cells (Figure 3G). Cycloheximide (CHX) chase assays revealed that activated mTORC2 significantly accelerated the degradation of GSDME‐N (Figure 3H). Similarly, RICTOR overexpression promoted GSDME‐N degradation, while RICTOR knockdown stabilized GSDME‐N levels (Figure 3I). Meanwhile, analysis of published public databases revealed a correlation between GSDME and RICTOR (Figure S3C,D). Using confocal laser scanning microscopy, we assessed the subcellular localization of GSDME‐N and RICTOR. Upon IR treatment, co‐localization reduced (Figure 3J). Conversely, knockdown of RICTOR/mTORC2 increased the proportion of Annexin V/PI double‐positive cells, indicating elevated pyroptosis (Figure 3K and Figure S3E). In contrast, overexpression of mTORC2‐related genes decreased the number of double‐positive cells (Figure 3L and Figure S3F). Collectively, these findings suggest that RICTOR/mTORC2 negatively regulates GSDME‐N stability, thereby modulating pyroptosis.

It is widely reported that radiotherapy can trigger multiple distinct programmed cell death pathways, including apoptosis and ferroptosis. However, in our SCLC model, we discovered that pyroptosis plays a predominant role. To rigorously exclude the potential contributions of ferroptosis and apoptosis, we performed the following investigations. First, morphological microscopic observation, along with cell viability and LDH release assays, consistently demonstrated that the ferroptosis‐specific inhibitor Ferrostatin‐1 (Fer‐1) exerted no protective effect on this cell death process (Figure S3G), and immunoblotting revealed that essential anti‐ferroptotic markers (GPX4 and SLC7A11) remained unchanged after mTORC2 impairment (Figure S3H). Additionally, the cell death was rescued by silencing GSDME and successfully re‐instated by introducing the active GSDME‐N domain (Figure 3K and Figure S3E), which firmly excludes ferroptosis since it is driven by lipid peroxidation and is strictly GSDME‐independent. Second, unlike the characteristic cell shrinkage and formation of apoptotic bodies seen in apoptosis, the impaired cells exhibited rapid swelling and distinctive large, spherical membrane blisters (ballooning) (Figure S3G). Immunoblotting revealed that GSDME silencing abolishes RICTOR‐knockdown‐induced GSDME‐N generation, molecularly confirming strictly GSDME‐dependent pyroptosis over classical apoptosis (Figure S3I). Functionally, this early membrane pore formation and rupture led to a massive release of intracellular macromolecules and ready PI dye uptake, contrasting sharply with the intact membranes and lack of leakage in early apoptosis (Figure S3J). Furthermore, unlike the immunologically silent nature of apoptosis, this cell rupture was accompanied by the robust release of pro‐inflammatory cytokines (such as IL‐1β) (Figure S3K). In conclusion, we demonstrate that RICTOR/mTORC2 inhibition specifically exacerbates GSDME‐mediated pyroptosis rather than apoptosis or ferroptosis.

### 2.4 RICTOR/mTORC2 Promotes Radioresistance in SCLC

**FIGURE 4:**
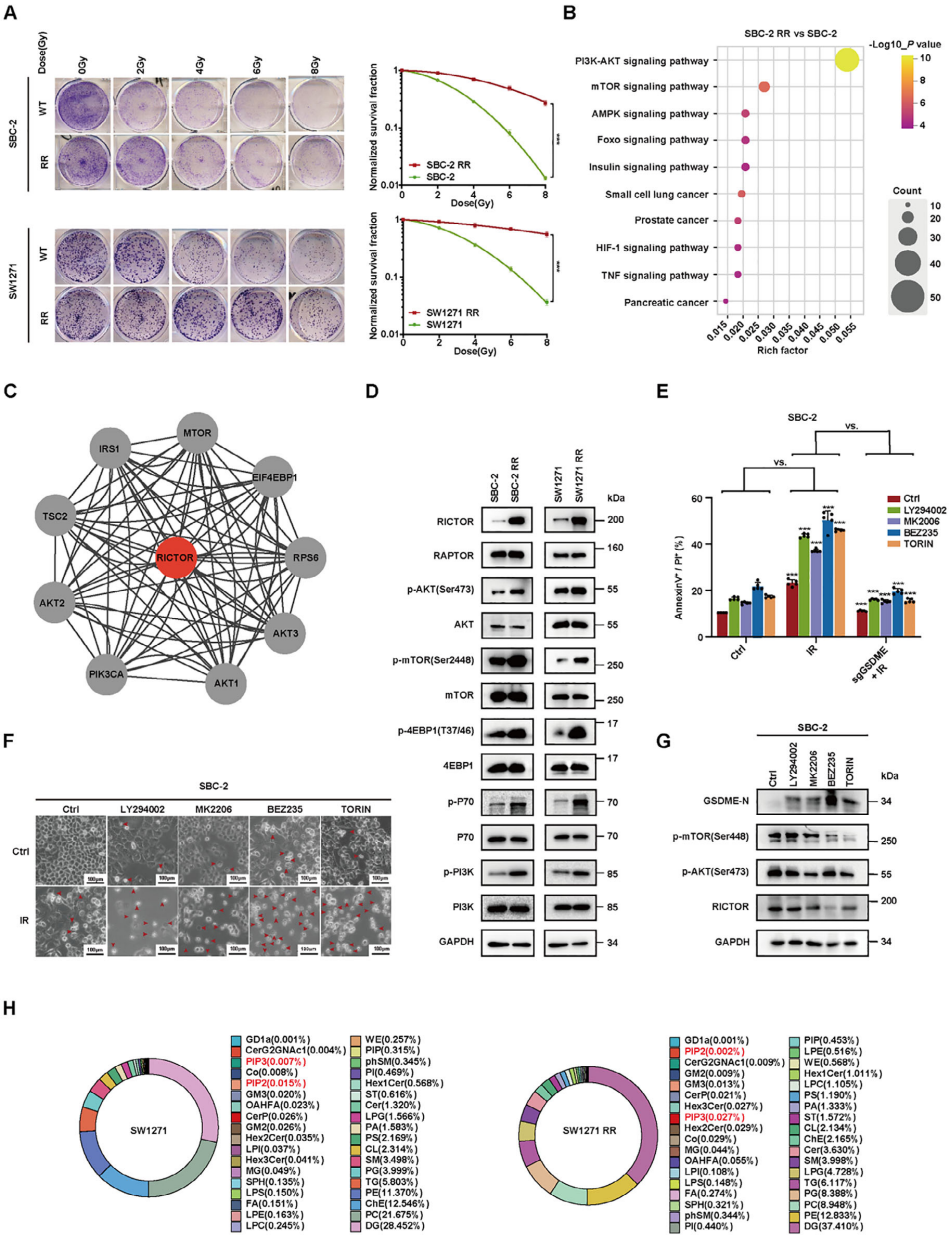
RICTOR/mTORC2 promotes radioresistance in SCLC. (A) Colony formation assays showing survival fractions of SBC‐2, SW1271, and their corresponding radioresistant derivatives (SBC‐2 RR and SW1271 RR) following radiation. (B) KEGG pathway enrichment analysis of upregulated genes in SBC‐2 RR cells compared to SBC‐2 cells. The top 10 enriched pathways are shown. (C) Protein‐protein interaction (PPI) network analysis of genes enriched in the pyroptosis pathway comparing SBC‐2 RR to SBC‐2, highlighting key downregulated components. (D) Validation of sequencing results by western blotting of selected genes in SCLC and radioresistant cell pairs. (E) Quantification of Annexin V^+^/PI^+^ SBC‐2 cells following treatment with the indicated kinase inhibitors (LY294002, MK2006, BEZ235, or TORIN) under basal conditions (Ctrl, IR, or sgGSDME + IR). Mean (*n* = 5) ± SD. (F) Phase‐contrast microscopy images showing pyroptotic morphological features in SCLC cells treated with IR and mTOR inhibitors. (G) Western blotting analysis of GSDME‐N expression after treatment with different PI3K/AKT/mTOR inhibitors. (H) Lipidomic analysis showing changes in membrane phosphoinositide content (PIP2 and PIP3) in SW1271 RR vs. SW1271 cells. Data are representative of three independent experiments in (A, D, E–G). Results are presented as mean ± SD; ^*^ *p* < 0.05, ^**^ *p* < 0.01, ^***^ *p* < 0.001, ns = not significant; *p* values were determined using two‐way ANOVA.

To investigate the mechanisms underlying radioresistance in SCLC, we established a radioresistant cell model by exposing SCLC cells to fractionated radiation (2 Gy per day), mimicking clinical radiotherapy protocols, until a cumulative dose of 50 Gy was achieved. Morphologically, the radioresistant cells exhibited increased size and granularity, markedly distinct from radiosensitive cells (Figure S4A–C). The establishment of this model was confirmed by clonogenic assays following graded radiation doses, which demonstrated increased survival of radioresistant cells compared to their parental counterparts (Figure 4A). To elucidate the molecular mechanisms involved, we conducted RNA‐seq analysis on parental and radioresistant cell lines (SBC‐2, SBC‐2 RR, SW1271, SW1271 RR). KEGG pathway enrichment analysis revealed significant activation of the PI3K‐AKT and mTOR signaling pathways in radioresistant cells (Figure 4B,C and Figure S4D,E). Given that the PI3K‐AKT pathway is known to activate both mTORC1 and mTORC2, this supports the notion that mTOR signaling contributes to radioresistance in SCLC. Western blot analysis confirmed elevated levels of RICTOR, phosphorylated AKT (Ser473), phosphorylated p70S6K, and phosphorylated 4EBP1 in the radioresistant cells, whereas the total protein of these molecules remained unchanged (Figure 4D). To assess the functional relevance of this signaling axis, we performed Annexin V/PI double‐staining assays under radiation exposure and in the presence of pharmacologic inhibitors: LY294002 (PI3K inhibitor), MK2206 (AKT inhibitor), BEZ235 (dual PI3K/AKT inhibitor), and TORIN (mTOR inhibitor). These inhibitors markedly increased cell death, indicating enhanced pyroptosis upon pathway inhibition, while GSDME knockdown suppressed this effect (Figure 4E and Figure S4F). Morphological analysis also confirmed the presence of typical pyroptotic features under these treatment conditions (Figure 4F and Figure S4G). Western blotting showed that these inhibitors significantly upregulated GSDME‐N protein levels, further supporting their role in facilitating pyroptosis (Figure 4G). Additionally, we observed increased expression of membrane‐bound GSDME‐N upon treatment with PI3K/AKT/mTOR inhibitors (Figure S4H). Mechanistically, PI3K activation converts phosphatidylinositol‐4,5‐bisphosphate (PIP2) into phosphatidylinositol‐3‐phosphate (PIP3) at the plasma membrane. Using lipid extraction and quantification, we found that PIP2 levels were reduced, while PIP3 levels were elevated in SCLC RR cells, indicating enhanced PI3K pathway activity (Figure 4H and Figure S4I). Since GSDME‐N preferentially binds to PIP2, reduced PIP2 availability may impair membrane localization of GSDME‐N, rendering it more susceptible to degradation and thereby attenuating pyroptosis [24].

### 2.5 RICTOR /mTORC2 Phosphorylates GSDME‐N to Inhibit Pyroptosis

**FIGURE 5:**
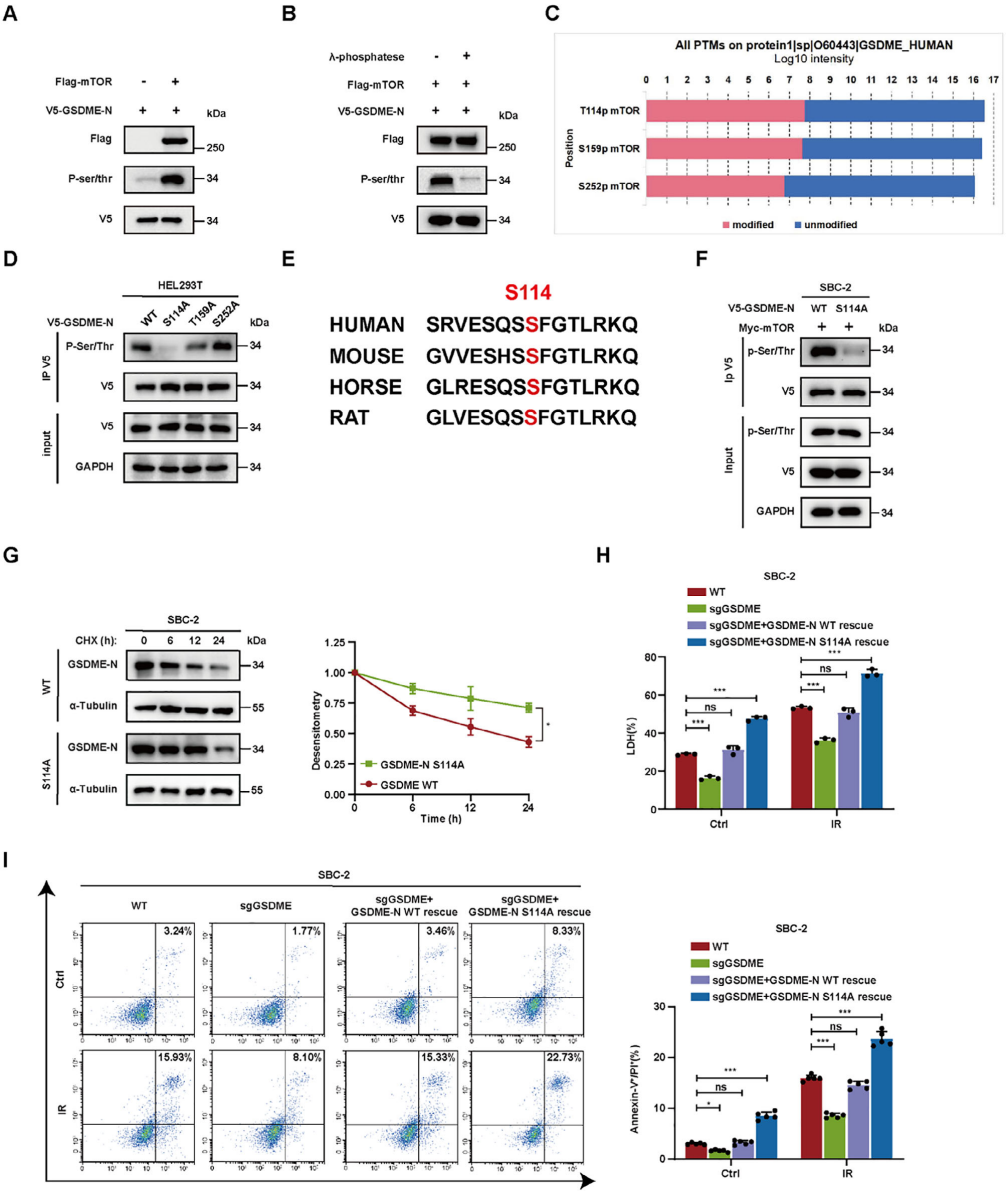
RICTOR/mTORC2 phosphorylates GSDME‐N to inhibit pyroptosis. (A, B) GSDME‐N was phosphorylated by mTOR in vitro kinase assay. V5‐GSDME‐N was transfected into 293T cells together with Flag‐mTOR or vector. The V5‐GSDME‐N and Flag‐mTOR were incubated with or without λ‐phosphatase and analyzed by in vitro kinase assay. (C) Potential phosphorylation sites on GSDME‐N were identified by mass spectrometry (MS). (D) Co‐IP analysis of phosphorylation levels after site‐directed mutagenesis of GSDME‐N. (E) Evolutionary conservation analysis of predicted GSDME‐N phosphorylation sites. Conserved phosphorylation residues are highlighted in red. (F) Co‐IP analysis assessing the effect of the S114 mutation on GSDME‐N phosphorylation in SCLC cells. (G) CHX chase assay comparing the protein stability of wild‐type GSDME‐N and phosphorylation‐deficient mutants (S114A). Plots showing the normalized GSDME‐N levels were also presented. (H, I) Flow cytometry (*n* = 5) and LDH release assays evaluating the functional impact of S114 mutation and restore plasmids in SBC‐2 and SBC‐2 IR cells. Data are representative of three independent experiments in (A, B, D, F, G–I). Results are presented as mean ± SD; ^*^ *p* < 0.05, ^**^ *p* < 0.01, ^***^ *p* < 0.001, ns = not significant; *p* values were determined using two‐way ANOVA.

Our previous results indicated that mTORC2 inhibits GSDME‐N‐mediated pyroptosis, particularly by impairing GSDME‐N membrane localization. Given that mTOR exhibits serine/threonine kinase activity [25], we hypothesized that mTORC2 might directly mediate GSDME‐N phosphorylation. To examine whether mTOR directly phosphorylated GSDME, we performed an in vitro kinase phosphorylation assay and found through Western blotting analysis using a general phospho‐serine/threonine antibody that the addition of the mTOR kinase significantly increased the phosphorylation of GSDME‐N (Figure 5A). To further confirm this finding, λ‐phosphatase (PPase) was added to the in vitro kinase assay system, and the signal of phosphorylation disappeared (Figure 5B). To validate these findings, MS identified several potential phosphorylation sites on GSDME‐N: S114, T159, S252 (Figure 5C). IP results showed that GSDME‐N was efficiently phosphorylated by mTOR, whereas a mutation at S114 (S114A) substantially reduced phosphorylation (Figure 5D). Sequence alignment of the Ser114‐containing motif revealed high evolutionary conservation across species, underscoring its potential functional significance (Figure 5E). Additionally, immunoprecipitation using pan‐serine/threonine phosphorylation antibodies further confirmed mTOR‐mediated phosphorylation at S114 (Figure 5F). We next assessed the effect of phosphorylation on GSDME‐N protein stability. Cycloheximide chase assays demonstrated that the phosphorylation‐deficient mutant (S114A) had a significantly longer half‐life compared to wild‐type GSDME‐N (Figure 5G). Following GSDME knockout in SBC‐2 cells, we conducted rescue experiments by re‐expressing either wild‐type GSDME‐N or the S114A mutant. LDH release assays and Flow cytometry analysis showed that cells expressing the S114A mutant exhibited significantly enhanced pyroptosis, compared to those expressing wild‐type GSDME‐N (Figure 5H,I). In summary, these findings demonstrate that mTORC2 phosphorylates GSDME‐N at S114, thereby reducing its protein stability and ultimately suppressing pyroptosis.

### 2.6 RICTOR/mTORC2 Regulates GSDME‐N Degradation via the Proteasomal Ubiquitin Pathway

**FIGURE 6:**
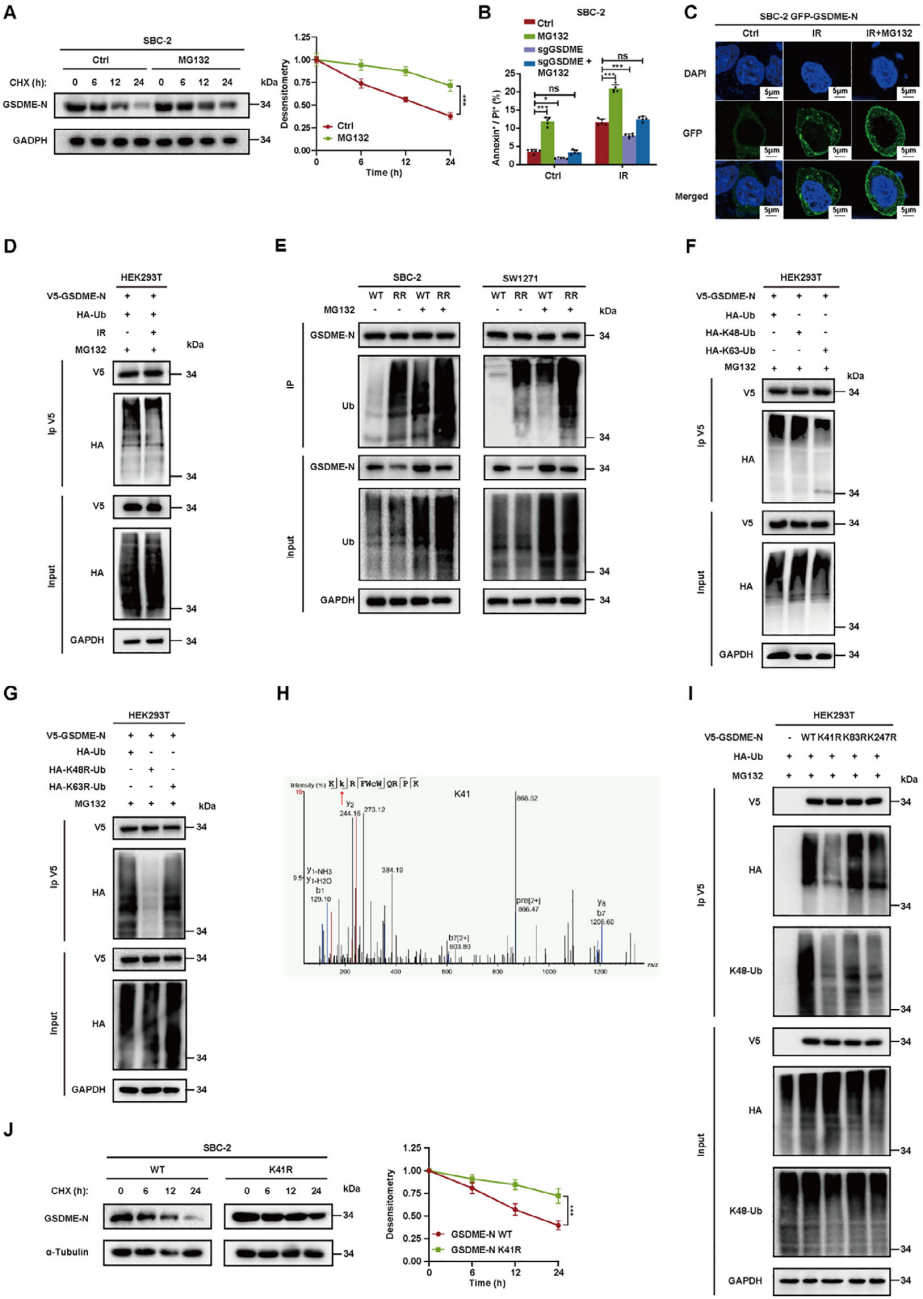
RICTOR/mTORC2 regulates GSDME‐N degradation via the proteasomal ubiquitin pathway. (A) CHX chase assays were performed in the presence of MG132 (proteasome inhibitor) to assess GSDME‐N degradation dynamics. Plots showing the normalized GSDME‐N levels were also presented. (B) Flow cytometry (Annexin V/PI) demonstrating the effect of MG132 treatment on pyroptosis under IR in SBC‐2 cells (*n* = 5). (C) Laser confocal observation was conducted on the membrane localization of GSDME‐N after the addition of MG132 and IR. (D) Co‐expression of GSDME‐N and HA‐tagged ubiquitin in 293T cells, followed by ubiquitination immunoprecipitation to assess ubiquitin modification. (E) Comparison of endogenous GSDME‐N ubiquitination levels in radioresistant vs. wild‐type SBC‐2 and SW1271 cells. (F) Ubiquitination immunoprecipitation analysis of 293T cells co‐expressing GSDME‐N with either K48‐only or K63‐only HA‐tagged ubiquitin to determine linkage type. (G) Co‐transfection of GSDME‐N with K48R or K63R ubiquitin mutants to verify the specific ubiquitin linkage involved in GSDME‐N degradation. (H) LC‐MS identified peptide sequences of GSDME‐N K41 ubiquitination sites (I) SDM of lysine residues followed by ubiquitination immunoprecipitation to validate functional ubiquitin modification sites. (J) CHX chase assays assessing the effect of K41 mutation on GSDME‐N protein stability. Plots showing the normalized GSDME‐N levels were also presented. Data are representative of three independent experiments in (A–G, I–J). Results are presented as mean ± SD; ^*^ *p* < 0.05, ^**^ *p* < 0.01, ^***^ *p* < 0.001, ns = not significant; *p* values were determined using two‐way ANOVA.

To investigate the degradation pathway of GSDME‐N, CHX chase assays showed that proteasome inhibitor (MG132) treatment markedly increased GSDME‐N protein levels, whereas autophagy lysosome inhibitor (BafA1) had only a minimal effect (Figure 6A and Figure S5A) [26]. The expression of GSDME‐N increased in a dose‐dependent manner with escalating concentrations of MG132, which revealed MG132 significantly inhibited the proteasomal degradation of GSDME‐N protein (Figure S5B). Furthermore, Flow cytometry analysis revealed that in SCLC cells, the proportion of Annexin V/PI double‐positive cells significantly increased after combined treatment with IR and MG132, compared to IR alone or MG132 alone. However, this elevation was partially reversed upon GSDME gene knockout, indicating that the synergistic effect strictly relied on the GSDME‐N‐mediated pyroptosis pathway (Figure 6B and Figure S5C). Laser confocal microscopy analysis revealed that the membrane signal intensity of GSDME‐N was significantly enhanced in cells treated with MG132 or MG132 in combination with IR compared to the control group (Figure 6C and Figure S5D). This series of results indicates that GSDME‐N is primarily degraded through the proteasome pathway.

To further explore the underlying mechanism, Co‐IP revealed that GSDME‐N was found to be ubiquitinated; however, ubiquitination levels were modestly reduced upon IR treatment, suggesting that IR may trigger early steps of pyroptosis by relieving this degradation (Figure 6D). In SCLC RR cells, the ubiquitination level of endogenous GSDME‐N was significantly elevated compared to SCLC WT cells, which may explain the reduced GSDME‐N expression observed in radioresistant cells (Figure 6E). Ubiquitin can form polyubiquitin chains through seven lysine (K) residues, with K48‐linked chains typically targeting proteins for degradation, whereas K63‐linked chains often regulate subcellular localization and signal transduction [27, 28]. To determine the specific ubiquitin linkage type on GSDME‐N, we conducted IP assays and demonstrated that K48‐linked ubiquitination of GSDME‐N was comparable to wild‐type, while K63‐linked ubiquitination was minimal (Figure 6F). To validate these results, the K48R significantly reduced GSDME‐N ubiquitination, whereas K63R had little effect by IP (Figure 6G), confirming that GSDME‐N undergoes K48‐linked ubiquitination, targeting it for proteasomal degradation and contributing to radioresistance in SCLC.

To identify the specific lysine residue(s) on GSDME‐N responsible for ubiquitination, we treated cells with MG132, enriched GSDME‐N via immunoprecipitation, and subjected the samples to LC‐MS analysis. Among the candidate sites, K41, K83, and K247 were identified as ubiquitination sites (Figure 6H). We subsequently generated point mutants at K41, K83, and K247. Notably, mutation at K41 resulted in a marked reduction in ubiquitination, as confirmed using both general and K48‐specific ubiquitin antibodies (Figure 6I). Finally, CHX chase experiments demonstrated that GSDME‐N K41 mutants exhibited enhanced protein stability compared to the wild‐type (Figure 6J).

### 2.7 RICTOR/mTORC2 Promotes CUL4B‐RBBP4 Binding to GSDME‐N, Leading to its Ubiquitin‐Mediated Degradation

**FIGURE 7:**
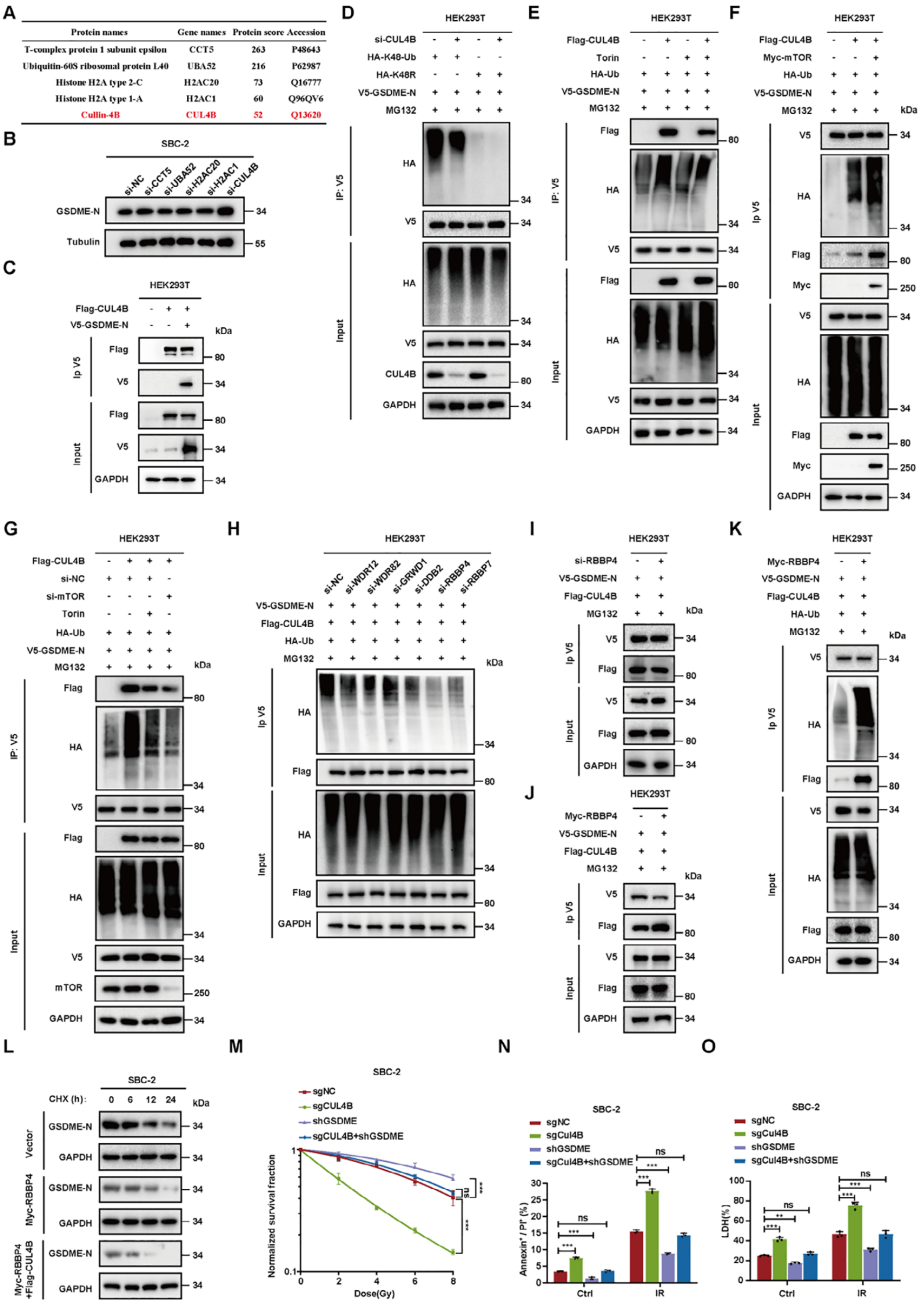
RICTOR/mTORC2 promotes CUL4B‐RBBP4 binding to GSDME‐N, leading to its ubiquitin‐mediated degradation. (A) List of candidate proteins from MS. (B) Western blotting showing the expression level of GSDME‐N in the SBC‐2 cells following siRNA‐mediated knockdown of the five candidate genes. (C) Co‐immunoprecipitation (Co‐IP) assay showing the interaction between co‐transfected CUL4B and GSDME‐N in 293T cells. (D) Cells were co‐transfected with V5‐GSDME‐N and HA‐tagged K48 ubiquitin (HA‐K48‐Ub) or mutant ubiquitin (HA‐K48R‐Ub) for in vivo ubiquitination assays. (E) Ubiquitination immunoprecipitation was used to detect the effect of CUL4B overexpression on GSDME‐N ubiquitination. (F,G) Ubiquitination and Co‐IP assays evaluating the impact of mTOR activity on CUL4B‐GSDME‐N interaction and GSDME‐N ubiquitination. (H) siRNA knockdown screening of candidate adaptor proteins identified by previous Co‐IP in GSDME‐N interaction. (I) Co‐IP assay in 293T cells co‐transfected with CUL4B and GSDME‐N, assessing the effect of RBBP4 knockdown on their interaction. (J) Co‐IP assay demonstrating direct interaction between overexpressed RBBP4 and GSDME‐N in 293T cells. (K) Ubiquitination immunoprecipitation was conducted to detect ubiquitination modification of GSDME‐N under conditions of RBBP4 overexpression. (L) Protein half‐life assay revealing enhanced degradation of GSDME‐N upon overexpression of RBBP4 alone or in combination with CUL4B. (M) Radiosensitivity assay of SBC‐2 cells with CUL4B knockout (sgCUL4B) or GSDME knockdown expression or both. (N) Flow cytometry analysis of cell death (Annexin V/PI) in SBC‐2 cells after IR (8 Gy), following CUL4B knockout or GSDME knockdown or both. (O) The LDH release assay was detected in SBC‐2 transfected with CUL4B knockout or GSDME knockdown or both. Data are representative of three independent experiments in (B–O). Results are presented as mean ± SD; ^*^ *p* < 0.05, ^**^ *p* < 0.01, ^***^ *p* < 0.001, ns = not significant; *p* values were determined using two‐way ANOVA.

Having confirmed that GSDME‐N undergoes K48‐linked ubiquitination, we sought to identify the E3 ubiquitin ligase complex responsible for its degradation. GSDME‐N was overexpressed in HEK293T cells, and interacting proteins were identified using immunoprecipitation combined with MS. Among the candidates, we identified CUL4B, a member of the Cullin‐RING E3 ligase family, as a potential interactor. Database analysis further supported CUL4B as the only candidate among the top five E3 ligases with a known association to the PI3K‐AKT‐mTORC2 signaling axis (Figure 7A and Figure S6A) [29]. To further verify the MS result, five E3 ligases were knocked down in SBC‐2 cells. Knockdown of CUL4B significantly increased GSDME‐N expression by Western blotting, suggesting its involvement in GSDME deubiquitination (Figure 7B). IP assays confirmed the interaction between CUL4B and GSDME‐N in HEK293T cells (Figure 7C). CUL4B knockdown significantly reduced GSDME‐N K48‐linked ubiquitination chains (Figure 7D). Conversely, CUL4B overexpression enhanced GSDME‐N K48‐linked ubiquitination. Crucially, targeted knockdown (si‐mTOR) or pharmacological inhibition (Torin) of mTOR not only disrupted the physical binding between CUL4B and GSDME‐N but also substantially attenuated CUL4B‐driven ubiquitination. Conversely, co‐overexpression of mTOR elevated this protein interaction and synergistically augmented GSDME‐N ubiquitination (Figure 7E–G). Furthermore, silencing endogenous CUL4B or mTOR effectively diminished the basal ubiquitination of GSDME‐N (Figure S6B). Together, these evidences definitively establish that mTOR promotes the recruitment of CUL4B to GSDME‐N, facilitating its ubiquitin‐mediated degradation.

Like other Cullin family ligases, CUL4B requires a substrate adaptor for specific protein recognition. By reviewing the literature and our MS data, we identified several WD40 domain‐containing proteins, known to function as adaptors, including 60 such candidates in the mammalian genome [30]. Among them, six classical CUL4B‐associated adaptor proteins were found to interact with GSDME‐N (Figure S6C,D). Using siRNA knockdown screening, we identified *RBBP4* as the key adaptor responsible for GSDME‐N recognition (Figure 7H). In a CUL4B‐GSDME‐N co‐expression system, *RBBP4* knockdown significantly impaired the interaction between CUL4B and GSDME‐N (Figure 7I). Conversely, *RBBP4* overexpression promoted its interaction with GSDME‐N (Figure 7J) and increased GSDME‐N ubiquitination (Figure 7K). Subsequently, we performed qPCR, which confirmed that knockdown of RBBP4 effectively downregulated its own expression but did not affect the mRNA level of GSDME (Figure S6E). Moreover, CHX chase experiments showed that *RBBP4* overexpression accelerated GSDME‐N degradation, and this effect was further enhanced when *CUL4B* and *RBBP4* were co‐expressed (Figure 7L and Figure S6F). These findings demonstrate that the CUL4B‐RBBP4 E3 ligase complex mediates the K48‐linked ubiquitination of GSDME‐N, promoting its proteasomal degradation.

To determine the role of CUL4B in SCLC radioresistance, in sgCUL4B cell models, shGSDME expression was restored via plasmid transfection. The CUL4B knockout significantly elevated radiosensitivity, the proportion of Annexin V/PI double‐positive cells, the release of LDH, and knockdown of GSDME in CUL4B‐knockout cells significantly reversed the effects, suggesting that CUL4B could regulate GSDME‐N‐mediated pyroptosis (Figure 7M–O and Figure S6G,H). In summary, our data support a model in which the CUL4B‐RBBP4 complex, regulated by RICTOR/mTORC2, mediates K48‐linked ubiquitination and degradation of GSDME‐N, thereby promoting radioresistance in SCLC.

### 2.8 Hyperactivated mTORC2 Leads to Radioresistance In Vivo and is Associated With Poor Prognosis in SCLC Patients

**FIGURE 8:**
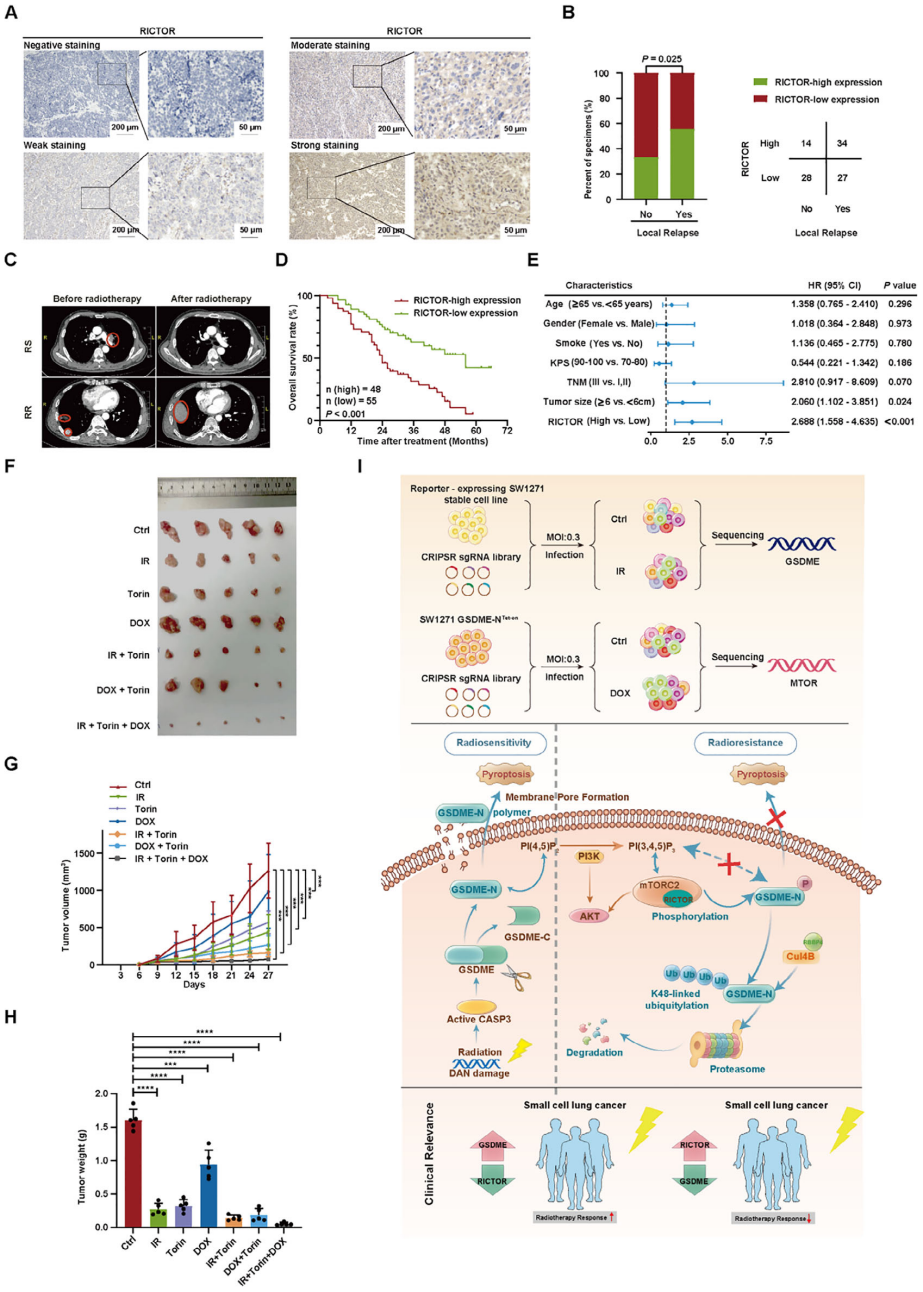
Hyperactivated mTORC2 leads to radioresistance in vivo and is associated with poor prognosis in patients with SCLC. (A) IHC staining of RICTOR in 103 SCLC tissues, categorized by staining intensity. Scale bar = 50 µm. (B) Association between RICTOR expression (IHC) and local relapse status in SCLC patients; *p*‐value determined by two‐tailed χ^2^ test. (C) Representative CT scans showing tumor regression in radiosensitive and tumor progression in radioresistant SCLC patients pre‐ and post‐radiotherapy. (D) Kaplan‐Meier analysis of overall survival (OS) based on RICTOR expression levels; *P*‐values calculated using the log‐rank test. (E) Forest plot from multivariate Cox regression analysis identifying significant prognostic variables in SCLC, including RICTOR. (F) Representative images of tumors excised at the end of the in vivo experiment. (G, H) Tumor volume and weight measurements of xenografts in nude mice treated with DOX, TORIN, IR, or their combinations. (I) Schematic diagram summarizing the proposed molecular model of RICTOR/mTORC2‐CUL4B‐GSDME‐N regulation in SCLC radioresistance. Data are representative of three independent experiments in (G, H). Results are presented as mean ± SD; ^*^ *p* < 0.05, ^**^ *p* < 0.01, ^***^ *p* < 0.001, ns = not significant; *p* values were determined using two‐way ANOVA.

To further assess the clinical relevance of RICTOR expression in SCLC, we performed IHC on tumor tissues from 103 SCLC patients. Based on staining intensity, samples were categorized into four groups: negative, weak, moderate, and strong expression (Figure 8A). Meanwhile, based on the clinical data, we found that locoregional tumor recurrence after radiotherapy in SCLC patients was significantly associated with high RICTOR expression (Figure 8B). Computed Tomography (CT) imaging demonstrated tumor shrinkage in radiosensitive patients and progression in radioresistant ones (Figure 8C). Additionally, Analysis of clinical outcomes revealed that high RICTOR expression was significantly associated with worse OS, PFS, and LRFS (Figure 8C and Figure S7A). Multivariate Cox regression analysis further confirmed that RICTOR expression level was an independent prognostic indicators in SCLC (Figure 8D, Figure S7B and Table S3). To functionally evaluate the effect of mTORC2 on SCLC radiosensitivity in vivo, Tumor volume measurements (volume and weight) and images of seven groups of dissected mice revealed that the combination of IR, DOX, and TORIN produced the strongest tumor growth inhibition (Figure 8E–G). Tissue proteins were extracted from these groups and analyzed by Western blotting to evaluate the expression levels of GSDME‐N and cleaved caspase‐3. The results demonstrated elevated protein expression of GSDME‐N and cleaved caspase‐3 in the IR, DOX, and TORIN groups compared to the Control group. The highest expression of GSDME‐N protein proved that the induced pyroptosis was the most obvious after DOX and IR combined with TORIN (Figure S7C). Meanwhile, serum LDH release levels of the seven groups of mice were detected before treatment, two weeks after treatment, and on the day of execution, and it was found that serum LDH concentration was highest after DOX and IR combined with TORIN group (Figure S7D). Collectively, these findings indicate that low RICTOR expression is associated with a favorable prognosis, and pharmacological inhibition of mTORC2 (via TORIN) can induce pyroptosis and enhance the radiosensitivity of SCLC. Mechanistically, activation of the mTORC2 signaling pathway promotes the recruitment of CUL4B ubiquitin ligase to GSDME‐N through specific phosphorylation at the S114 residue. This interaction facilitates K48‐linked polyubiquitination and proteasomal degradation of GSDME‐N, thereby suppressing pyroptosis and contributing to radioresistance in SCLC (Figure 8H).

## 3 Discussion

Radioresistance remains a major obstacle in the treatment of SCLC and is a key contributor to treatment failure and poor patient prognosis [31, 32, 33]. Despite its clinical significance, the molecular mechanisms underlying SCLC radioresistance remain incompletely understood. In this study, by establishing radioresistant SCLC cell models and conducting a genome‐wide CRISPR activation screen combined with multi‐omics analysis, we identified that defects in pyroptosis serve as a key mechanism underlying radioresistance in SCLC, and the sustained activation of mTORC2 plays a critical role in this process. Our findings demonstrate that mTORC2 contributes to resistance by suppressing GSDME‐N‐mediated pyroptosis, a form of programmed inflammatory cell death. Mechanistically, MS and Co‐IP assays revealed that mTORC2 phosphorylates GSDME‐N at S114. This phosphorylation facilitates the recruitment of the E3 ubiquitin ligase complex CUL4B‐RBBP4, which mediates K48‐linked ubiquitination at lysine 41 (K41) of GSDME‐N. The resulting polyubiquitination targets GSDME‐N for proteasomal degradation, thereby preventing its pyroptotic function. Functional experiments in both in vitro cell lines and in vivo xenograft models confirmed that knock down of RICTOR restores GSDME‐N expression and function, leading to enhanced pyroptosis and significantly improved radiotherapy sensitivity. Moreover, clinical analysis of SCLC patient cohorts revealed that low RICTOR expression was significantly associated with greater radiosensitivity and prolonged survival. These findings establish the RICTOR/mTORC2‐CUL4B‐GSDME axis as a central regulatory pathway in SCLC radioresistance.

Pyroptosis is a form of programmed cell death mediated by inflammasomes and is critically dependent on the GSDM and inflammatory caspase protein families [34]. In brief, pyroptosis is initiated when activated caspases cleave GSDMs, releasing their N‐terminal domains [6]. These N‐terminal fragments bind to membrane lipids and form pores in the plasma membrane, disrupting osmotic balance and causing cell swelling and eventual lysis through membrane rupture [35, 36]. Therefore, GSDMs serve as the primary executioners of pyroptosis [37].

Historically, only a limited number of studies have explored the role of the GSDM family in pyroptosis [38, 39]. Under chemotherapeutic conditions, caspase‐3 cleaves GSDME, releasing the GSDME‐N fragment, which induces pyroptosis by perforating the cell membrane [11]. Similarly, caspase‐8 cleaves GSDMC, triggering pyroptosis in triple‐negative breast cancer cells [40]. However, these studies primarily focused on full‐length GSDME or its cleavage into the N‐terminal fragment, while post‐translational regulation—particularly ubiquitination—of GSDME‐N has not been well characterized. In this study, we uncovered a novel mechanism by which GSDME‐N is rendered unstable within tumor cells and undergoes ubiquitin‐mediated proteasomal degradation.

The mTOR signaling pathway plays a central role in integrating extracellular signals to regulate a wide array of cellular processes, including metabolism, angiogenesis, cell survival, growth, and proliferation [41]. Its involvement in therapeutic resistance is well‐established. For instance, AKT mutations have been shown to accelerate the cell cycle and promote the rapid repair of radiation‐induced DNA damage, thereby contributing to radioresistance [42, 43]. Additionally, these mutations promote the degradation of FOXO transcription factors, thereby inhibiting apoptosis initiation [44]. mTOR signaling is closely associated with the PI3K/AKT pathway, and its dysregulation is frequently implicated in cancer progression and resistance to therapy [45, 46]. In our study, we observed that neither PIK3CA nor AKT altered GSDME‐N degradation levels. This led us to focus on mTOR, and specifically on its two major regulatory complexes, mTORC1 (RAPTOR) and mTORC2 (RICTOR). Our findings demonstrated that mTORC2, but not mTORC1, modulates GSDME‐N protein degradation. Although mTORC2 is traditionally known for its role in growth factor signaling, its direct involvement in cell death regulation has not been previously reported.

In our study, we observed that loss of RICTOR, a core component of mTORC2, significantly enhanced GSDME‐N‐mediated pyroptosis and altered its protein stability without affecting its mRNA levels. Our co‐immunoprecipitation experiments showed that mTOR can directly bind to GSDME‐N, and this binding is primarily achieved through the mTORC2 component RICTOR. Further confocal microscopy experiments also revealed co‐localization of RICTOR and GSDME‐N on the membrane. Collectively, these experiments suggest that mTORC2 directly interacts with GSDME‐N to mediate pyroptosis blockade. Given that mTOR is a classic serine/threonine kinase, this distinct post‐translational phenotype naturally prompted the hypothesis of a direct phosphorylation event. This hypothesis is strongly supported by recent structural and biochemical advances demonstrating that mTORC2 functions as an active kinase that directly phosphorylates multiple distinct substrates to regulate their protein stability and subcellular localization, such as AKT [47], SGK1 [48], and PKC [49]. Subsequently, we substantiated this hypothesis using in vitro kinase and phosphatase assays, which provided direct biochemical proof that mTORC2 phosphorylates GSDME‐N. To pinpoint the precise molecular interface, we utilized mass spectrometry coupled with point mutation screening and evolutionary conservation analysis, successfully identifying S114 as the critical functional phosphorylation site. Finally, we closed the logical loop through protein half‐life tracking and functional rescue assays, demonstrating unequivocally that preventing phosphorylation at S114 (via S114A mutation) prolongs the half‐life of GSDME‐N and intensifies pyroptosis. In conclusion, we uncover a novel mechanism wherein mTORC2 directly phosphorylates GSDME‐N, promoting its degradation through the ubiquitin‐proteasome pathway, thereby inhibiting pyroptosis. This discovery highlights a previously unrecognized mode of mTORC2 activation and function. In radioresistant cells, activation of the PI3K‐mTOR signaling pathway affects GSDME‐N in two ways. First, it changes the lipid composition of the cell membrane by converting PIP2 into PIP3. Since GSDME family proteins prefer to insert into PIP2‐rich membranes, this conversion prevents GSDME‐N from being recruited to the membrane. Second, mTORC2 can directly bind to GSDME‐N in the cytoplasm and phosphorylate it, which leads to the degradation of the protein. As a result, free GSDME‐N becomes more likely to be broken down.

The Cullin protein family, the largest class of E3 ubiquitin ligases, comprises seven major isoforms: Cullin 1, 2, 3, 4A, 4B, 5, and 7 [50]. CUL4B, in particular, regulates substrate stability by recognizing specific proteins and targeting them for ubiquitin‐mediated degradation [51]. Known CRL4B substrates include ERα, cyclin E, TOPO I, WDR5, PRX III, TSC2, Jab1/CSN5, P53, RGS2, and HUWE1. In our study, we identified GSDME‐N as a novel substrate of the CUL4B E3 ligase complex, demonstrating that CUL4B facilitates radioresistance in SCLC by promoting GSDME‐N ubiquitination and degradation. Importantly, we showed that ubiquitylated GSDME‐N regulated pyroptosis, and stabilization of GSDME‐N restored pyroptotic activity, thereby reversing radioresistance in SCLC cells.

This study has several limitations that warrant consideration. Although our analyses identified high RICTOR expression as significant predictors of poor prognosis in SCLC, and confirmed their roles as independent prognostic factors, these conclusions were drawn from a relatively small patient cohort. Therefore, larger, multicenter clinical studies will be necessary to validate and generalize these findings. Additionally, the in vivo therapeutic models used in this study do not fully reflect the genomic complexity and heterogeneity of human SCLC. To enhance translational relevance, future studies should utilize more physiologically representative systems, such as patient‐derived organoids and patient‐derived xenograft (PDX) models, to rigorously assess the therapeutic potential of targeting the RICTOR‐CUL4B‐GSDME axis in SCLC.

## 4 Conclusion

In this study, we conducted a CRISPR activation screen and identified GSDME as a key modulator of radiosensitivity in SCLC cells. Further investigations revealed that RICTOR/mTORC2 contributes to radioresistance by inhibiting GSDME‐N‐mediated pyroptosis. Mechanistically, mTORC2 phosphorylates GSDME‐N, facilitating the recruitment of the CUL4B‐RBBP4 ubiquitin ligase complex, which mediates K48‐linked ubiquitination and subsequent proteasomal degradation of GSDME‐N. This degradation suppresses pyroptosis and promotes resistance to radiotherapy. Importantly, inhibition of mTORC2 or overexpression of GSDME‐N restored pyroptotic activity and enhanced radiosensitivity in both in vitro and in vivo models. These findings identify the RICTOR/mTORC2‐CUL4B‐GSDME axis as a novel mechanism of radioresistance in SCLC and suggest it may serve as a promising therapeutic target for overcoming radioresistance.

## 5 Experimental Section

### 5.1 Cell Lines

The human embryonic kidney cell line HEK293T was obtained from the American Type Culture Collection (Manassas, VA, USA). Human SCLC cell lines SBC‐2 and SW1271 were purchased from Cobioer Corporation (Nanjing, Jiangsu, China). All cell lines were maintained in either Roswell Park Memorial Institute (RPMI) 1640 medium (Invitrogen, Carlsbad, CA, USA) or Dulbecco's Modified Eagle Medium (DMEM) (Gibco, Grand Island, NY, USA), supplemented with 10% fetal bovine serum (FBS) (Gibco, Grand Island, NY, USA). All cell lines were routinely tested for mycoplasma contamination and authenticated using short tandem repeat (STR) profiling.

### 5.2 Plasmid Construction

GSDME (P34161), RPTOR independent companion of mTOR complex 2 (RICTOR) (P41727), retinoblastoma binding protein 4 (RBBP4) (G36752) were obtained from MiaoLingBio (Wuhan, Hebei, China). Regulatory associated protein of mTOR complex 1 (RAPTOR) (1859) was obtained from BioVector NTC (Beijing, China). N‐terminal fragment of GSDME (GSDME‐N) was cloned into the pLVX ‐TRE vector with a V5 tag. Site‐directed mutagenesis was performed to generate lysine‐to‐arginine substitutions (K41R, K83R, K247R) in V5‐GSDME‐N, and various serine/threonine‐to‐alanine substitutions (S114A, T159A, S252A) in V5‐GSDME‐N. All constructs were generated using the ClonExpress II One Step Cloning Kit (C112‐01) or ClonExpress MultiS One Step Cloning Kit (C113‐01) (Vazyme, Nanjing, Jiangsu, China), and verified by DNA sequencing. Hemagglutinin‐tagged ubiquitin (HA‐Ub), wild‐type ubiquitin variants (K48Ub, K63Ub), and their lysine‐to‐arginine mutants (K48R, K63R) were generously provided by Professor Xiaofeng Zhu (Sun Yat‐sen University Cancer Center, SYSUCC; Guangzhou, Guangdong, China).

### 5.3 Cell Transfection and Lentiviral Infection

Lentiviral packaging was performed using the packaging plasmid psPAX2 and the envelope plasmid pMD2.G. The transfer plasmid pLenti‐TRE‐GSDME‐N, along with the packaging vectors, was co‐transfected into HEK293T cells. After 48 h, the culture supernatant containing lentiviral particles was harvested and used to infect SBC and SW1271 cells. Infected cells were selected with puromycin (1 µg/mL). To establish a doxycycline (DOX)‐inducible Tet‐on expression system, GSDME‐N was cloned into the pLenti‐Tet vector (Addgene). Following lentiviral transduction, cells were selected with blasticidin S (15 µg/mL), and single‐cell clones were isolated to ensure uniform induction. GSDME‐N expression was subsequently induced by treatment with DOX. For RNA interference, small interfering RNA (siRNA) oligonucleotides were synthesized by GenePharma (Suzhou, Jiangsu, China). A scrambled siRNA duplex was used as a negative control. Knockdown efficiency was assessed by qRT‐PCR and Western blotting. The sequences of siRNAs are provided in Table S1. Single guide RNA (sgRNA) sequences targeting GSDME or CUL4B were designed using an online sgRNA design platform (https://benchling.com). The corresponding sgRNA sequences (5’‐3’) are listed in Table S1.

### 5.4 CRISPR/Cas9 Screen

For genome‐wide CRISPR activation screening, 1 × 10^8^ SW1271 cells were transduced with the human CRISPR activation library (Calabrese P65‐HSF) at a multiplicity of infection (MOI) of 0.3. After 5 days of puromycin selection, the surviving cells were divided into two groups: one group was exposed to IR, and the other remained untreated. After the cells undergo pyroptosis, the surviving cells are maintained in culture for 7 days, followed by cell collection and DNA extraction. Genomic DNA was extracted from both groups, and PCR amplification was performed to construct sequencing libraries. The resulting data were analyzed using the Model‐based Analysis of Genome‐wide CRISPR‐Cas9 Knockout (MAGeCK, version 0.5.9.2; https://sourceforge.net/projects/mageck/) and MAGeCKFlute pipelines developed by the Dana‐Farber Cancer Institute (Boston, MA, USA) [52, 53]. A secondary CRISPR screen was conducted using SW1271 cells stably expressing GSDME‐N^Tet‐on^ plasmid. These cells were transduced with the same CRISPR activation library at an MOI of 0.3, followed by puromycin selection for 5 days. To maintain adequate library representation, cells were expanded to achieve at least 500‐fold coverage of the gRNA library. The GSDME‐N^Tet‐on^ cells were then split into two groups, with one treated with DOX and the other left untreated. After the cells undergo pyroptosis, the surviving cells are maintained in culture for 7 days, followed by cell collection and DNA extraction. Sequencing and data analysis were performed as described for the initial screen.

### 5.5 Construction of Radioresistant SCLC Cells

SCLC cell lines SBC‐2 and SW1271 were cultured in 75 cm^2^ flasks. Once cells adhered to the flask surface, they were exposed to a single dose of 2 Gy IR. The irradiated cells were then returned to the incubator, and their morphology and growth were closely monitored. After recovery, cells were subjected to repeated 2 Gy irradiation doses until a cumulative dose of 50 Gy was achieved. The surviving cell population was considered to be radioresistant and was maintained for further experiments.

### 5.6 Clonogenic Assay

SBC‐2 and SW1271 cells were seeded into six‐well plates at a density of 1000 cells per well. Following cell attachment, the cultures were irradiated with gradient doses (2, 4, 6, and 8 Gy). Cells were then incubated for 7–14 days until visible colony formation was observed in the untreated control group. The culture medium was discarded, and residual serum was washed away with PBS. Cells were fixed using paraformaldehyde (PFA, Boster, Wuhan, Hubei, China) and stained with 0.5% crystal violet solution (BIOISCO, Lianyungang, Jiangsu, China). Colonies consisting of more than 50 cells were counted manually. Cell survival curves were then generated.

### 5.7 Morphological Assessment of Pyroptosis

To distinguish morphological features of pyroptosis, apoptosis, and viable cells, SBC‐2 cells were seeded at a density of 1 × 10^5^ cells per 35 mm confocal dish. Static cell images were acquired using an Olympus IX73 fluorescence microscope (Olympus, Tokyo, Japan). For dynamic live‐cell imaging, GFP‐labeled SBC‐2 cells (1 × 10^5^) were seeded into 35 mm glass‐bottom culture dishes. Time‐lapse imaging was performed using the CV1000 confocal scanner system (Yokogawa, Tokyo, Japan). Pyroptotic, apoptotic, and surviving cells were quantified and analyzed using CV1000 imaging software.

### 5.8 RNA Extraction, Reverse Transcription, and Quantitative PCR (qPCR)

Total RNA was extracted from tissues and cells using TRIzol reagent (EZBiosciences, Roseville, MN, USA) according to the manufacturer's protocol. Reverse transcription was performed using the ReverTra Ace qPCR RT Kit (#FSQ‐101, TOYOBO, Shanghai, China). qPCR was conducted with TB Green Premix Ex Taq (#RR820A, Takara, Tokyo, Japan). PCR cycling conditions were as follows: initial activation of HotStarTaq DNA polymerase (Qiagen, Hilden, Germany) at 95°C for 15 min, followed by 45 cycles of denaturation at 94°C for 15 s, annealing at 55°C for 30 s, and extension at 70°C for 30 s. Fluorescence data were collected during the extension phase. Relative gene expression levels were calculated using the comparative threshold cycle (2^−ΔΔCt^) method. Statistical significance was evaluated using Student's *t*‐test. Each experiment was repeated three times. GAPDH mRNA was used as an internal control. Primer sequences are provided in Table S1.

### 5.9 Western Blotting Analysis

SCLC cells were lysed on ice using RIPA buffer (Cell Signaling Technology, Boston, MA, USA) supplemented with protease inhibitors, followed by sonication to extract total protein. Equal amounts of protein (15 µg per lane) were resolved by SDS‐PAGE on 8%–15% Bis‐Tris polyacrylamide gels (Beyotime, Shanghai, China; Invitrogen, Grand Island, NY, USA) and electrophoresed at 150 V for 1.5 h. Proteins were transferred onto polyvinylidene fluoride (PVDF) membranes (Merck Millipore, Billerica, MA, USA) at 110 mA for 3–5 h. Membranes were blocked and probed with the indicated primary antibodies (Table S2), followed by appropriate secondary antibodies. Immunoreactive bands were visualized using the ChemiDoc Imaging System (Bio‐Rad, Hercules, CA, USA).

### 5.10 Co‐Immunoprecipitation (Co‐IP) and Mass spectrometry (MS)

SCLC and HEK293T cells were lysed on ice using IP lysis buffer (MedChemExpress, MCE, NJ, USA) supplemented with protease inhibitors and phosphatase inhibitor cocktail (Selleck, Houston, TX, USA). For IP, 1 mg of total protein lysate was incubated overnight at 4°C with 1–2 µg of specific antibodies, followed by an additional 1‐h incubation with Protein A/G agarose beads. The antibodies used for IP are listed in Table S2. Immunoprecipitated proteins were subjected to Western blotting or MS analysis (Wininovate Bio, Shenzhen, China). For phosphosite identification, enriched phosphopeptides were isolated via TiO_2_ affinity chromatography or phospho‐specific immunoprecipitation. Phosphorylation sites were determined using MaxQuant software (Martinsried, Munich, Germany). For ubiquitination site mapping, proteins were digested with trypsin/Lys‐C, and ubiquitinated peptides were enriched using anti‐diGly remnant antibodies. Ubiquitinated lysine residues were identified through the detection of the characteristic Gly‐Gly (diGly) remnant motif using FragPipe software developed by the Nesvizhskii Lab (University of Michigan, Ann Arbor, MI, USA).

### 5.11 In Vitro Kinase Assay

The kinase reaction system was assembled on ice by combining the following components in order: 40 µL of kinase reaction buffer, 1–5 µL of the purified kinase (e.g., 0.1–1 µg), 2–5 µL of the substrate protein/peptide, and 1–5 µL of ATP to a final concentration ranging from 1 to 200 µm. Appropriate controls were included: a positive control using a known phosphorylatable substrate and negative controls such as reactions omitting the kinase, omitting the substrate, or employing a kinase‐dead mutant. After thorough mixing, the reaction mixtures were incubated at 37°C for a duration between 5 and 60 min. The reactions were terminated by adding an equal volume of 2 × SDS loading buffer, followed by boiling at 95°C for 5 min, or alternatively, by adding EDTA to a final concentration of 10 mm to chelate Mg^2+^. Phosphorylation was subsequently detected by separating the kinase and substrate proteins via SDS‐PAGE, transferring them onto a membrane, and probing with a phosphorylation‐specific antibody via western blot analysis.

### 5.12 Flow Cytometry

Following the indicated treatments, SCLC cells were harvested, washed with phosphate‐buffered saline (PBS), and collected into centrifuge tubes. SCLC cells were resuspended in 500 µL of staining buffer, followed by incubation with Annexin V/PI staining solution (KeyGEN, Nanjing, Jiangsu, China) according to the manufacturer's instructions. The cells were incubated in the dark at room temperature (25°C) for 5–15 min. and were analyzed using a Beckman CytoFLEX flow cytometer (Beckman Coulter, Brea, CA, USA).

### 5.13 Lactate Dehydrogenase (LDH) Activity Test

To assess LDH release as an indicator of cell membrane integrity, culture supernatants were collected and centrifuged at 3000 × g for 5 min at 4°C. The LDH working solution was freshly prepared by mixing 1× 2‐p‐iodophenyl‐3‐nitrophenyl tetrazolium chloride / 5‐bromo‐4‐chloro‐3‐indolyl phosphate (INT/BCIP stock solution; Roche, Indianapolis, IN, USA), lactic acid, and enzyme solution in a 1:1:1 ratio. Subsequently, 60 µL of culture medium supernatant from each well was transferred to a new 96‐well plate and mixed with 30 µL of LDH working solution. The mixture was incubated at room temperature in the dark for 30 min. Absorbance was measured at 490 nm using a microplate reader (BioTek, Winooski, VT, USA), and LDH activity was quantified accordingly.

### 5.14 Enzyme‐Linked Immunosorbent Assay (ELISA)

The concentrations of human IL‐1β and IL‐6 in the culture supernatant were measured using ELISA kits (MM‐0181H2 and MM‐0049H2, respectively; MEIMIAN, Yancheng, Jiangsu, China). Supernatants were collected from cells treated with 8 Gy IR and from untreated control cells. Assays were performed in accordance with the manufacturer's protocols.

### 5.15 Immunofluorescence and Confocal Microscopy

Cells were first washed with PBS and fixed with 4% PFA for 10 min at room temperature. After fixation, cells were blocked with blocking buffer (Beyotime, Haimen, Nantong, China) for 1 h at room temperature and subsequently incubated overnight at 4°C in the dark with primary antibodies (Table S2). The next day, cells were washed three times with washing buffer (Beyotime, Haimen, Nantong, China) for 5 min each at room temperature. Samples were then incubated with fluorescently labeled secondary antibodies (Abclonal, Wuhan, Hubei, China) for 1 h at room temperature in the dark, followed by three additional washes. Nuclear staining was performed using DAPI (Beyotime, Haimen, Nantong, China). Cells were stored at 4°C in the dark until imaging. Fluorescent images were captured using a laser scanning confocal microscope (IX83, FV1000, Olympus, Tokyo, Japan). Quantification of fluorescence intensity and colocalization analysis were performed using ImageJ software (NIH, Bethesda, MD, USA).

### 5.16 Membrane Protein Extraction

SBC‐2 cells (5 × 10^7^) were collected by centrifugation at 600 × g for 5 min at 4°C after PBS washing. The supernatant was discarded, and membrane protein extraction reagent A (Beyotime, Shanghai, China) was added to the cell pellet. The suspension was transferred into a glass homogenizer and homogenized 30 times. The homogenate was then centrifuged at 700 × g for 10 min at 4°C, and the supernatant—representing the cytoplasmic protein fraction—was collected into a fresh centrifuge tube. The remaining pellet was resuspended in membrane protein extraction reagent B (Beyotime) and centrifuged at 14 000 × g for 5 min at 4°C. The resulting supernatant constituted the membrane protein fraction, which was used for downstream analysis.

### 5.17 Untargeted Lipidomics

For untargeted lipidomics analysis, the lipid extracts from SBC‐2, SBC‐2 RR, SW1271, and SW1271 RR cells were resuspended in 200 µL Eppendorf tubes and submitted to Shanghai Applied Protein Technology Co., Ltd. (Shanghai, China) for LC‐MS analysis. Lipids were separated on a Waters ACQUITY PREMIER CSH C18 column (1.7 µm, 2.1 × 100 mm; Waters Corporation, Milford, MA, USA). Chromatographic separation was achieved using mobile phase A (acetonitrile: water = 6:4, v/v) and mobile phase B (acetonitrile: isopropanol = 1:9, v/v), with a flow rate of 300 µL/min at a column temperature of 45°C. The gradient was programmed as follows: 30% mobile phase B for 2 min, ramping to 100% B over 23 min, then returning to 30% B in 1 min and equilibrated for an additional 9 min. Samples were placed in an autosampler maintained at 10°C and injected in a randomized sequence to minimize signal drift. Lipid detection was performed using a Thermo Scientific Q Exactive mass spectrometer (Thermo Fisher Scientific, Waltham, MA, USA) equipped with an electrospray ionization (ESI) source. LipidSearch 4.0 software (Thermo Fisher Scientific) was used for peak detection, internal standard calibration, and lipid identification.

### 5.18 Construction of In Vivo Tumor Growth Models

All animal procedures were approved by the Institutional Animal Care and Use Committee of Sun Yat‐sen University Cancer Center (SYSUCC) (Approval No. L025501202205009). BALB/c nude mice (4 weeks old) were purchased from GemPharmatech (Nanjing, Jiangsu, China) and housed under specific pathogen‐free (SPF) conditions. A total of 1 × 10^6^ SBC‐2‐GSDME‐N^Tet‐on^ cells suspended in PBS were subcutaneously injected into each mouse to establish a subcutaneous xenograft tumor model. Once tumors became palpable, mice were randomly assigned to either four groups (Control [no treatment], 0.2% DOX [HYD8960, HUAYUN, Guangzhou, China], 8 Gy irradiation [IR], and 0.2% DOX + IR) or seven groups (Control, IR, 0.2% DOX, 0.2 mg/mL TORIN [S2827, Selleck, Shanghai, China], IR + 0.2 mg/mL TORIN, 0.2% DOX + TORIN, and IR + 0.2% DOX + 0.2 mg/mL TORIN). Tumor volume and body weight were monitored every 2–3 days. On day 30, mice were euthanized by cervical dislocation. Mice were also humanely euthanized when tumor diameters exceeded 20 mm or tumor burden surpassed 10% of body weight. Excised tumor tissues were fixed, paraffin‐embedded, and subjected to immunohistochemistry (IHC) analysis.

### 5.19 Clinical Specimens

This study was approved by the Institutional Ethical Review Board of SYSUCC (Approval No. SL‐G2022‐122‐1). A total of 103 paraffin‐embedded tumor specimens were obtained from pathologically confirmed, non‐metastatic limited‐stage SCLC patients who had received either definitive radiotherapy or combined chemoradiotherapy. The inclusion criteria are patients with limited‐stage, non‐metgetastatic small cell lung cancer (SCLC) who are receiving radiotherapy or chemoradiotherapy. Exclusion criteria for our study included: creatinine clearance < 60 mL/min; Karnofsky Performance Status (KPS) score < 70; Subjects with incomplete documentation or follow‐up attrition. All patients provided written informed consent for the use of their tissue samples and clinical data for research purposes. Eligible cases were identified from January 1, 2015 to December 31, 2018, with histopathologically confirmed diagnoses. All patients provided written informed consent for the use of their tissue samples and clinical data for research purposes. Among patients who received radiotherapy, treatment response was categorized as follows: those achieving complete response (CR) or partial response (PR) were classified as radiosensitive, while those with progressive disease (PD) or stable disease (SD) were considered radioresistant. Our research design adhered to the REMARK (REporting recommendations for tumour MARKer prognostic studies) framework for standardized reporting in cancer biomarker investigations.

### 5.20 IHC Evaluation

IHC staining was performed on formalin‐fixed, paraffin‐embedded sections of SCLC tissue samples. Briefly, tissue sections were deparaffinized in xylene, rehydrated through a graded ethanol series, washed, and subjected to antigen retrieval using citrate buffer in a microwave oven. Endogenous peroxidase activity was quenched with 3% hydrogen peroxide. Antigen retrieval was further enhanced via high‐temperature EDTA treatment, followed by overnight incubation with primary antibodies at 4°C (Table S2). Images were captured using the AxioVision Rel. 4.6 computerized image analysis system (Carl Zeiss, Oberkochen, Baden‐Württemberg, Germany). Staining intensity was scored on a 4‐point scale: 0 (negative), 1 (weak), 2 (moderate), and 3 (strong). The percentage of positively stained tumor cells was scored as: 1 (<10%), 2 (10%–35%), 3 (35%–70%), and 4 (>70%). The immunoreactive score (IRS) was calculated by multiplying the intensity score by the percentage score, yielding a value between 0 and 12. Two pathologists, blinded to clinical information, independently reviewed and scored the samples according to the IRS scoring system [52]. IRS ≥ 6 were divided into high expression, while IRS below 6 were divided into low expression. For survival analysis, Kaplan‐Meier survival curves were generated to assess overall survival (OS), local relapse‐free survival (LRFS), and progression‐free survival (PFS). Statistical differences were assessed using log‐rank tests, and Cox proportional hazards models (α = 0.05) were applied using the coxph function in the R survival package.

### 5.21 RNA sequencing (RNA‐seq) Analysis

Total RNA was extracted from radiosensitive and radioresistant SCLC cell lines and tissue samples and subjected to RNA‐seq by Lianchuan Corporation (Hangzhou, Zhejiang, China). Differentially expressed genes (DEGs) were defined as those exhibiting an absolute log_2_ fold change (|log_2_FC|) > 1 with FDR‐adjusted *p* < 0.05. Functional enrichment analyses of DEGs were conducted using Gene Ontology (GO) terms and Kyoto Encyclopedia of Genes and Genomes (KEGG) pathways via the clusterProfiler toolkit (v4.6.2) [54]. Protein interaction network (PPI) analysis integrates RNA‐seq data to identify functionally interconnected hub genes or pathways, revealing key regulatory modules underlying observed transcriptional changes. Pathways or biological processes with a statistical significance threshold of *p* < 0.05 were considered significantly enriched.

### 5.22 Analyses of TCGA Data

Gene expression profiles were retrieved from the TCGA database. Expression values were normalized to Transcripts Per Million (TPM) and log_2_‐transformed for subsequent analyses. Correlation analyses between gene expression levels were performed using either Pearson or Spearman correlation coefficients, depending on the distribution of the data, to evaluate potential expression relationships between genes.

### 5.23 Statistical Analysis

All statistical analyses were performed using GraphPad Prism 9.5 (GraphPad Software, La Jolla, CA, USA). Data are presented as mean ± standard deviation (SD) unless otherwise specified. Group comparisons were conducted using the unpaired two‐tailed Student's *t*‐test or one‐way analysis of variance (ANOVA), as appropriate. Correlations were evaluated using the Pearson's χ^2^ test. A *p*‐value < 0.05 was considered statistically significant in all analyses.

## Author Contributions

Q.Q.X., C.M.S., S.X.Z., and R.L. contributed equally to this work. M.C., Q.Q.X., and X.L. conceived the experiments. Q.Q.X., Z.S.L., S.X.Z., and C.M.S. carried out and analyzed the data for most of the in vitro experiments. C.F.W., R.L., and Q.W.L. collected clinical data and performed the IHC experiments. M.C., Q.Q.X., X.L., L.L., and S.X.Z. designed and performed the animal experiments. C.M.S., X.L., and Q.Q.X. designed and performed LC‐MS/MS analysis. Y.Y.C., L.L., Z.S.L., and Q.W.L. helped with the data analyses. Q.Q.X., R.Z.C., and X.L. wrote and revised the manuscript. X.L., Q.Q.X., S.X.Z., and M.C. supervised the study. All authors reviewed and discussed the final version of the paper.

## Ethics Statement

Experiments regarding clinical samples were performed with the approval of the Institutional Ethical Review Boards of SYSUCC (Approval No. SL‐G2022‐122‐01). All clinical samples were collected from patients with written informed consent for the use of tissues and clinical data. All the animal experiments were approved by the Institutional Animal Care and Use Committee of SYSUCC (approval number L025501202205009).

## Conflicts of Interest

The authors declare no conflicts of interest.

## Supporting information




**Supporting File 1**: advs75844‐sup‐0001‐SuppMat.docx.

**Supporting File 2**: advs75844‐sup‐0002‐blotts.zip.

## Data Availability

The data that support the findings of this study are available from the corresponding author upon reasonable request.
