# Supplementary material for: mTORC2 Phosphorylation of GSDME‐N Drives Cullin4B‐Mediated Proteasomal Degradation to Suppress Pyroptosis and Confer Radioresistance in Small Cell Lung Cancer
Source: Adv Sci (Weinh). 2026 May 27:e75844. Online ahead of print. doi: 10.1002/advs.75844 (PMC13335795; doi:10.1002/advs.75844)
Supplement: Supplementary file 1 — Supporting File 1: advs75844‐sup‐0001‐SuppMat.docx. [file ADVS-9999-e75844-s002.docx]

**Supporting information**

**mTORC2 Phosphorylation of GSDME-N Drives Cullin4B-Mediated Proteasomal Degradation to Suppress Pyroptosis and Confer Radioresistance in Small Cell Lung Cancer**

Qing-qing Xu^1,2,3,†^, Ci-ming Sun^1,2,3,†^, Sui-xian Zhang^1,2,3,†^, Rui Li^1,2,3,†^, Chen-fei Wu^1,2,3^, Zai-shan Lin^1,2,3^, Li Li^1,2,3^, Run-zhe Chen^1,2,3^, Qi-wen Li^1,2,3^, Yuan-yuan Chen^1,2,3,*^, Xuan Li^1,2,3,*^, Ming Chen^1,2,3,*^

^1^State Key Laboratory of Oncology in South China, Guangdong Key Laboratory of Nasopharyngeal Carcinoma Diagnosis and Therapy, Guangdong Provincial Clinical Research Center for Cancer, Sun Yat-sen University Cancer Center, Guangzhou, 510060, GuangDong, P. R. China.

^2^Department of Radiation Oncology, Sun Yat-sen University Cancer Center, Guangzhou, 510060, GuangDong, P. R. China.

^3^United Laboratory of Frontier Radiotherapy Technology of Sun Yat-sen University & Chinese Academy of Sciences Ion Medical Technology Co, Guangzhou, 510060, GuangDong, P. R. China.

^†^These authors contributed equally: Qing-qing Xu, Ci-ming Sun, Sui-xian Zhang, Rui Li.

***Corresponding authors**:

Ming Chen: [chenming@sysucc.org.cn](mailto:chenming@sysucc.org.cn)

Department of Radiation Oncology, State Key Laboratory of Oncology in South China, Collaborative Innovation Center for Cancer Medicine, Sun Yat-sen University Cancer Center, 651 Dongfeng Road East, Guangzhou 510060, China; United Laboratory of Frontier Radiotherapy Technology of Sun Yat-sen University & Chinese Academy of Sciences Ion Medical Technology Co., Ltd, Guangzhou 510060,

P. R. China;

Xuan Li: [lixuan@sysucc.org.cn](mailto:lixuan@sysucc.org.cn)

State Key Laboratory of Oncology in South China, Collaborative Innovation Center for Cancer Medicine, Sun Yat-sen University Cancer Center, 651 Dongfeng Road East, Guangzhou 510060, China;

Yuan-yuan Chen: [chenyy2@sysucc.org.cn](mailto:chenyy2@sysucc.org.cn)

Department of Radiation Oncology, State Key Laboratory of Oncology in South China, Collaborative Innovation Center for Cancer Medicine, Sun Yat-sen University Cancer Center, 651 Dongfeng Road East, Guangzhou 510060, China;

**Supplementary Figures**


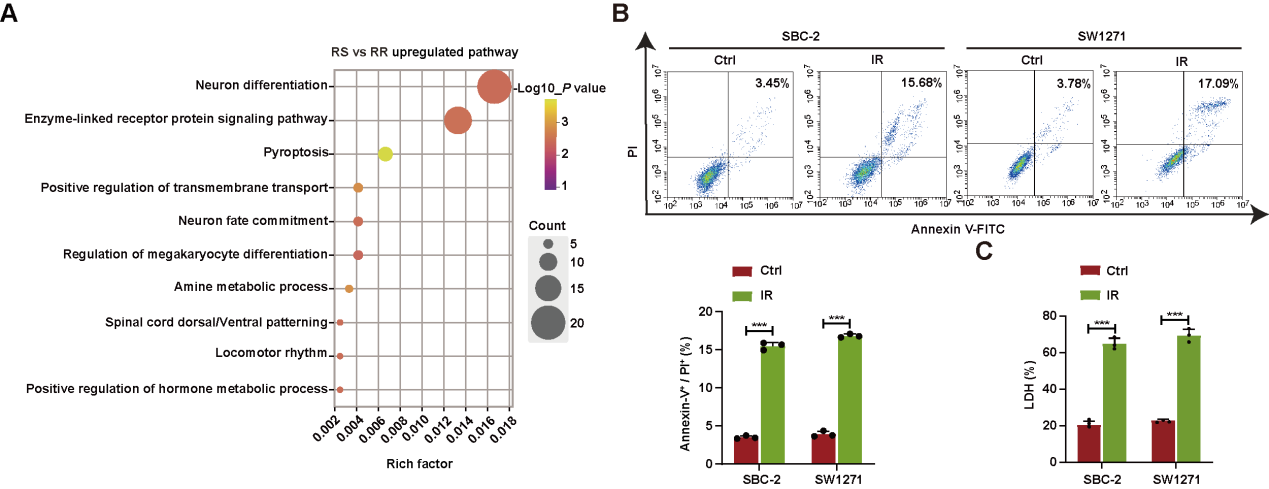


**Supplementary Figure S1 CRISPR/Cas9 screen identifies GSDME-mediated pyroptosis as a potential modulator of radioresistance in SCLC.** (A) Top 10 enriched pathways identified from differentially expressed genes using the GO Biological Process 2023 database via Enrichr. (B) Flow cytometry analysis of cell death levels (Annexin V/PI) in SBC-2 and SW271 cell lines with or without irradiation. (C) Lactate dehydrogenase (LDH) activity assay measuring LDH release levels in SBC-2 and SW1271 cell lines after irradiation. Data are representative of three independent experiments in B-C. Results are presented as mean ± SD; **p* < 0.05, ***p* < 0.01, ****p* < 0.001, ns = not significant; *p* values were determined using Student’s t-test.


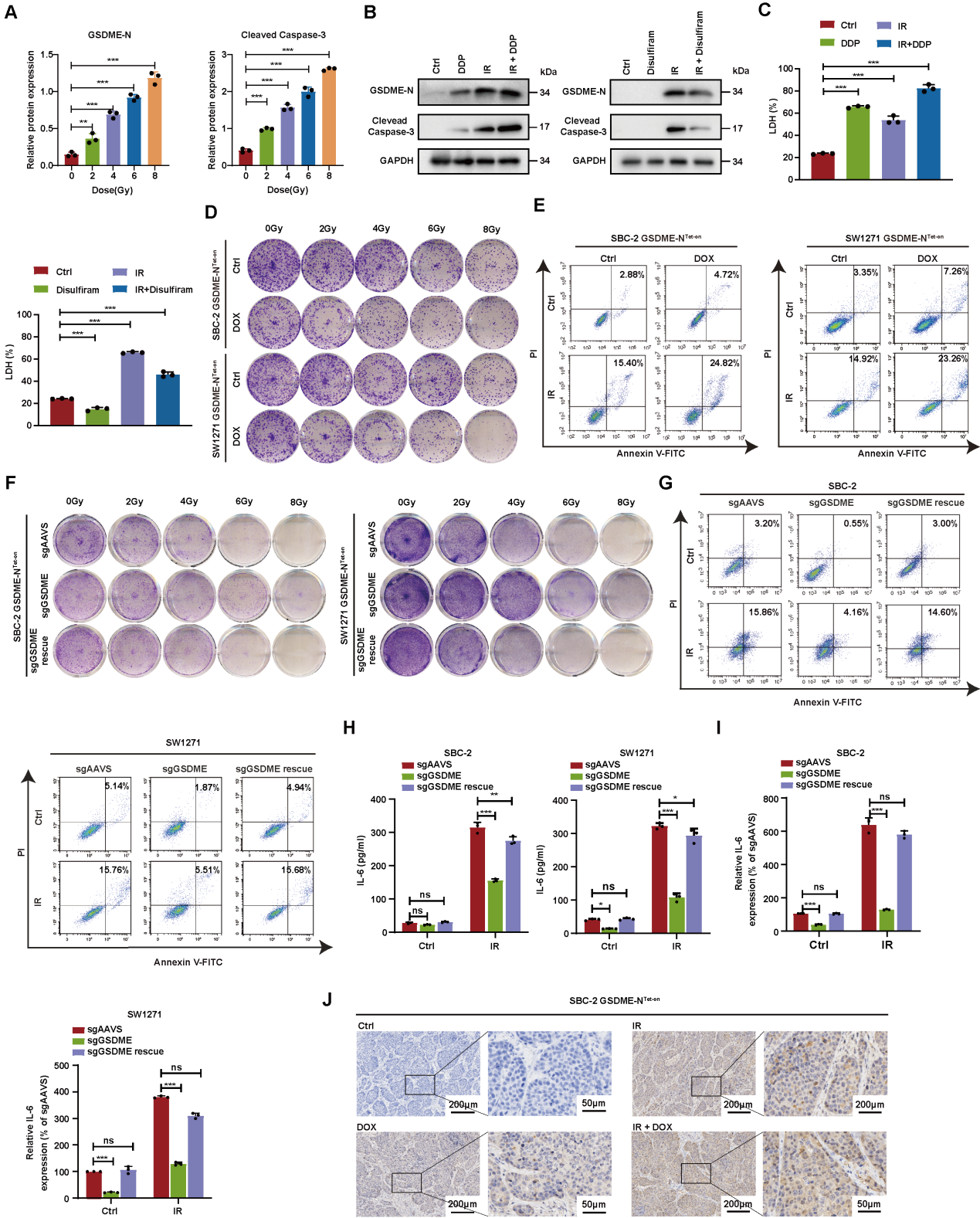


**Supplementary Figure S2. GSDME-N-mediated pyroptosis inhibited radioresistance in SCLC.** (A) Densitometric quantification of GSDME-N and Cleaved Caspase-3 relative protein levels in cells exposed to a radiation dose gradient (0, 2, 4, 6, 8 Gy). (B-C) Western blotting analysis of GSDME-N and Cleaved Caspase-3 and quantification of LDH release in SCLC cells treated with IR alone, IR combined with Cisplatin (DDP) or Disulfiram. (D) Representative colony formation assays image of SBC-2 GSDME-N^Tet-on^ and SW1271 GSDME-N^Tet-on^ cells following with or without DOX. (E) Flow cytometry assays showing increased pyroptosis following GSDME-N overexpression in SBC-2 GSDME-N^Tet-on^ and SW1271 GSDME-N^Tet-on^ cells. (F) Representative colony formation assays images of GSDME knockdown and restoration in SCLC cell. (G) Flow cytometric analysis of Annexin V/PI double-positive cells following GSDME knockdown and restoration in SCLC cell. (H) ELISA analysis of IL-6 in SCLC cells with GSDME knockdown and restoration in SCLC cell. (I) Quantitative PCR showing mRNA levels of IL-6 after GSDME modulation in SCLC cell. (J) Representative IHC images in xenograft tumor tissues from each treatment group (n = 5). Data are representative of three independent experiments in A to J. Results are presented as mean ± SD; **p* < 0.05, ***p* < 0.01, ****p* < 0.001, ns = not significant; *p* values were determined using one way ANOVA in A and C, Two way ANOVA in H and I.


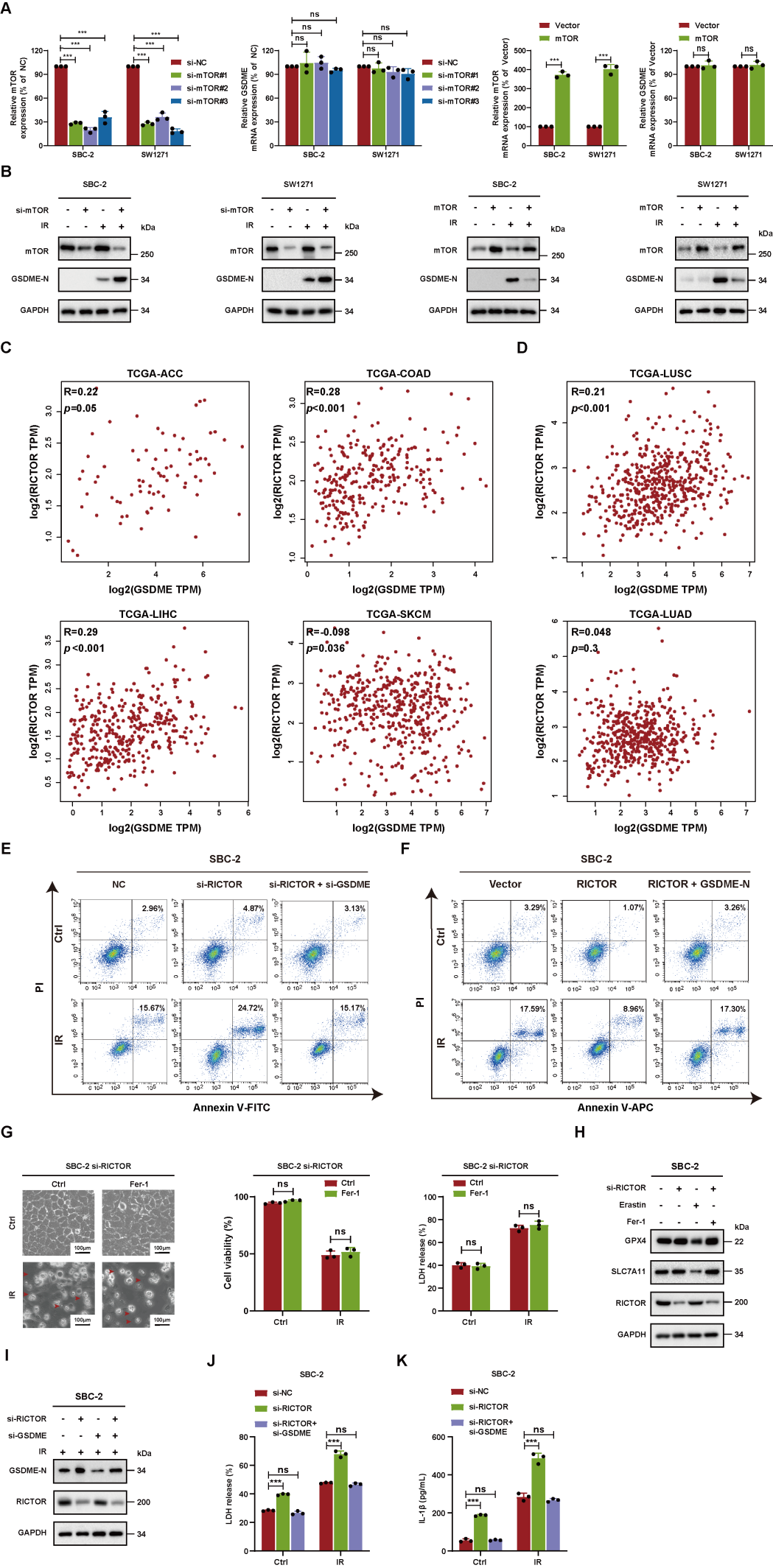


**Supplementary Figure S3. CRISPR screen reveals RICTOR/mTORC2 as a key regulator of GSDME-N-mediated pyroptosis.** (A) qRT-PCR was performed to assess transcriptional involvement and to confirm knockdown and overexpression efficiency of GSDME and mTOR. (B) SBC and SW1271 cells were transfected with mTOR-targeting siRNAs or mTOR overexpression plasmids for 48 h; total protein extracts were analyzed by immunoblotting. (C-D) Scatter plots depicted the correlation between log2-transformed GSDME TPM and RICTOR TPM across different TCGA cancer datasets. Pearson correlation coefficients (R) and corresponding *p*-values are presented to assess the strength and significance of the associations. (E-F) Flow cytometry analysis assessing the impact of knockdown or overexpression of mTOR, RICTOR on GSDME-N-mediated pyroptosis in SCLC cells. (G) Phase-contrast microscopy images showing morphological features in SBC-2 cells (left), Cell viability (medium) and LDH release assay (right) in control and RICTOR-knockdown cells treated with or without the ferroptosis inhibitor Ferrostatin-1 (Fer-1) (10 μM) at 48 hours. Scale bar, 100 μm. (H) Western blot analysis of the anti-ferroptotic markers GPX4 and SLC7A11 in SBC-2 cells following RICTOR knockdown. (I) Western blot analysis of GSDME-N in SBC-2 cells transfected with siNC, siRICTOR, or siRICTOR + siGSDME under irradiation (IR) conditions. (J) LDH release assay of SBC-2 cells transfected with siNC, siRICTOR, or siRICTOR + siGSDME in the presence or absence of irradiation (IR). (K) ELISA quantification of IL-1β levels released into the culture supernatant from the indicated groups. Data are representative of three independent experiments in A, B, E-K. Results are presented as mean ± SD; **p* < 0.05, ***p* < 0.01, ****p* < 0.001, ns = not significant; *p* values were determined using one-way ANOVA in A (left), Student’s t-test in A (right), two-way ANOVA in G, J and K; n = 3 independent experiments.


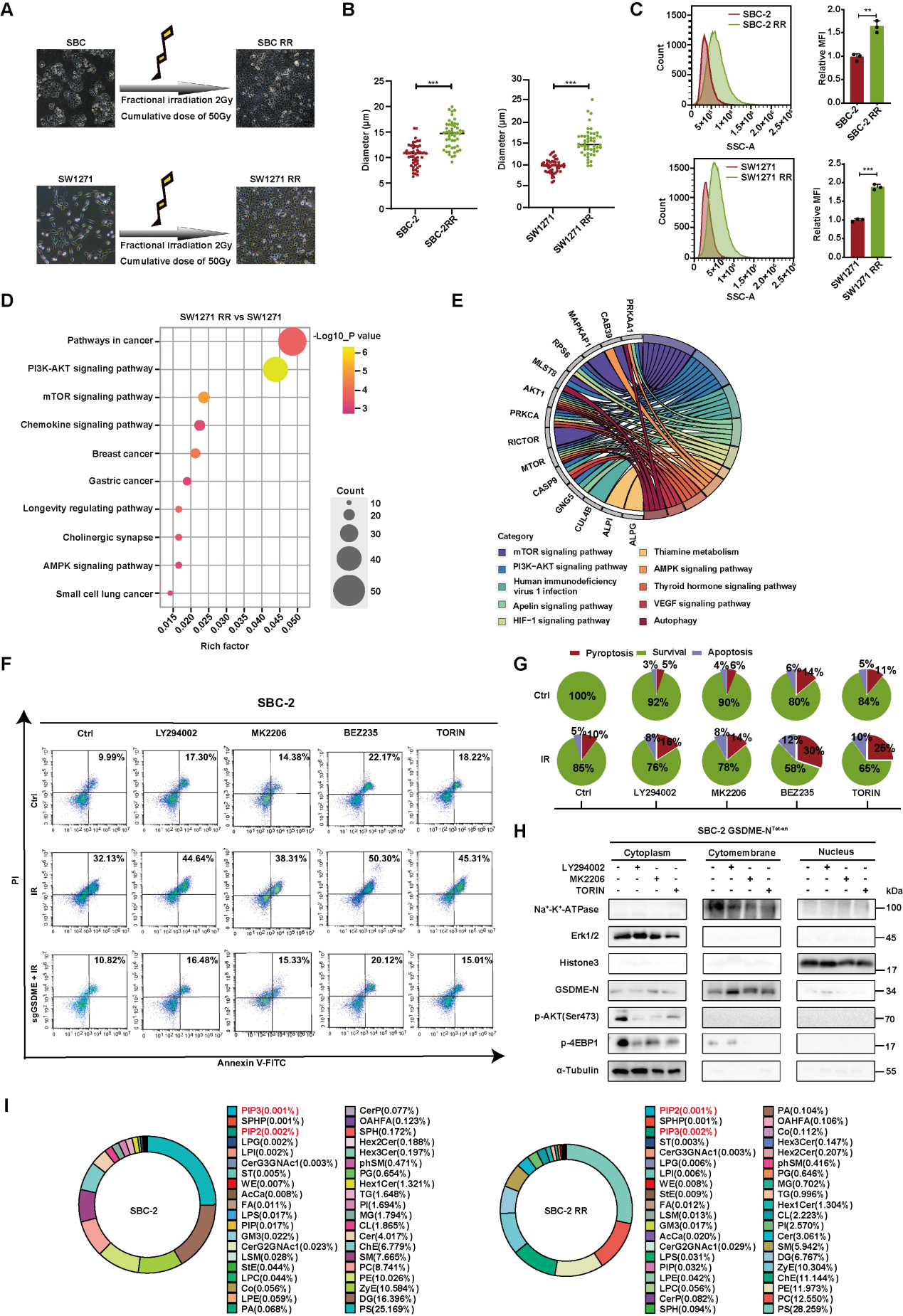


**Supplementary Figure S4. RICTOR/mTORC2 promotes radioresistance in SCLC.** (A) Schematic illustration of the strategy used to generate radioresistant (RR) SCLC cells lines. (B-C) Cell size was quantified by measuring cell diameter (n = 50) using ImageJ software, and granularity was assessed via Flow cytometry through analysis of side scatter (SSC) signal intensity in SCLC WT and RR cells. (D-E) KEGG pathway enrichment analysis of upregulated genes in SW1271 RR cells compared to SW1271 cells with three biological duplicates. The top 10 enriched pathways are shown. (F) Annexin V/PI double staining was used to assess the effects of IR, GSDME-N knockout, or mTOR inhibition (alone and in combination) on cell death in SCLC cells (n = 5). (G) Quantification of pyroptotic, apoptotic, and surviving cell populations treated with IR and PI3K/AKT/mTOR inhibitors. (H) Membrane protein extraction assays were conducted to detect membrane-associated GSDME-N expression in SBC-2 cells under inhibitor treatment. (I) Lipidomic analysis showing changes in membrane phosphoinositide content (PIP2 and PIP3) in SBC-2 RR versus SBC-2 cells. Data are representative of three independent experiments in A to C, F to H. Results are presented as mean ± SD; **p* < 0.05, ***p* < 0.01, ****p* < 0.001, ns = not significant; *p* values were determined using Student’s t-test in B-C.


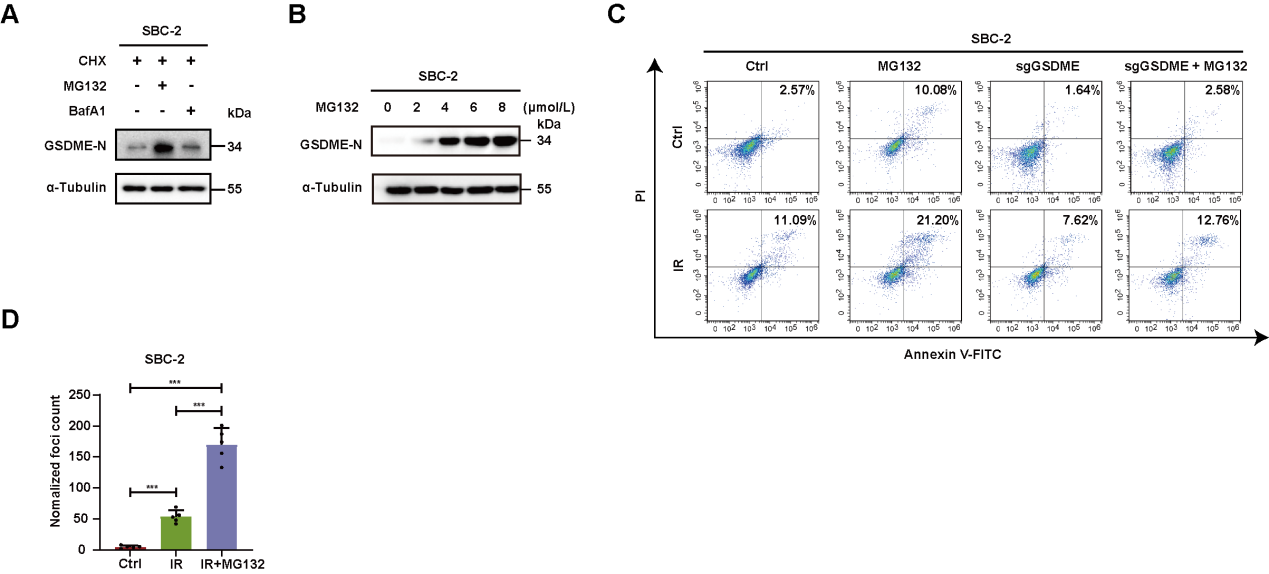


**Supplementary Figure S5. RICTOR/mTORC2 regulates GSDME-N degradation via the proteasomal ubiquitin pathway.** (A) CHX protein half-life assays and western blotting were performed to evaluate the effect of MG132 (proteasome inhibitor) and BafA1 (lysosome inhibitor) on GSDME-N degradation in SCLC cells. (B) Western blot analysis of GSDME-N levels in cells treated with increasing concentrations of MG132 (0, 2, 4, 6, 8 μmol/L). (C) Annexin V/PI double staining showing pyroptotic cell death in SBC-2 cells treated with MG132 and/or IR (n = 5). (D) The quantitative statistical graph of confocal immunofluorescence images showing membrane localization of GSDME-N after the addition of MG132 and IR. Data are representative of three independent experiments in A-D. Results are presented as mean ± SD; **p* < 0.05, ***p* < 0.01, ****p* < 0.001, ns = not significant; *p* values were determined using two-way ANOVA in D.


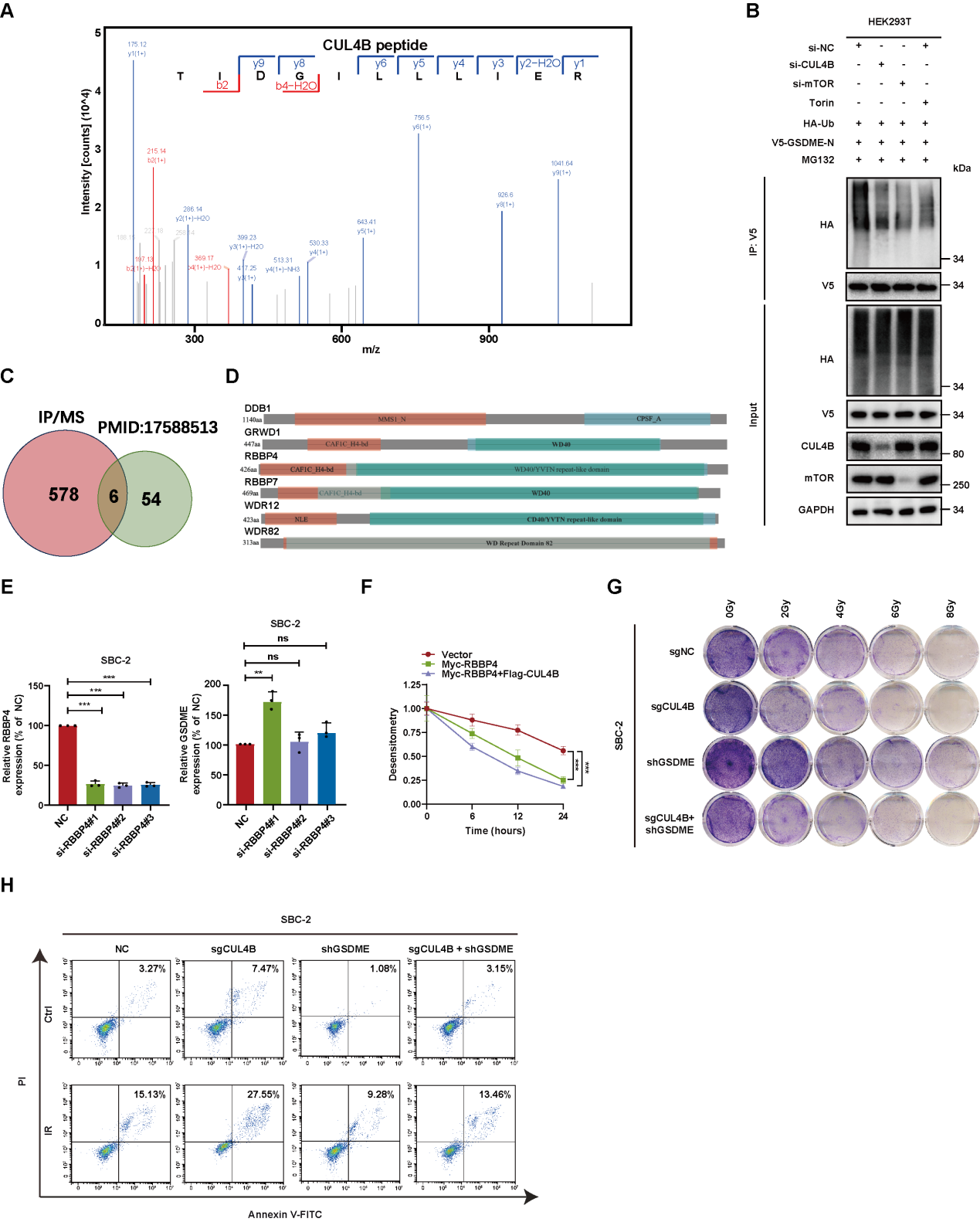


**Supplementary Figure S6. RICTOR/mTORC2 promotes CUL4B-RBBP4 binding to GSDME-N, leading to its ubiquitin-mediated degradation.** (A) Identification of potential E3 ligases mediating K48-linked ubiquitination of GSDME-N by cross-referencing UniProt and ubiquitin-related databases. (B) Immunoprecipitation (IP) and immunoblot analysis of GSDME-N ubiquitination levels in SCLC cells following pharmacological inhibition with Torin or genetic knockdown using small interfering RNA (siRNA). (C) Venn diagram showing the overlap between mass spectrometry data and literature-reported CUL4B-interacting proteins potentially linked to GSDME-N degradation. (D) Domain structures of six canonical CUL4B-associated adaptor proteins predicted to interact with GSDME-N. (E) qPCR analysis of RBBP4 and GSDME mRNA expression following RBBP4 knockdown in SCLC cells. (F) Plots showing the normalized GSDME-N levels upon overexpression of RBBP4 alone or in combination with CUL4B. (G) Representative colony formation assays images in SCLC cell with CUL4B knockout (sgCUL4B) or GSDME knockdown expression or both. (H) Annexin V/PI double staining showing cell death rates in SBC-2 cells with CUL4B knockdown or GSDME knockdown or both. Data are representative of three independent experiments in B, E to H. Results are presented as mean ± SD; **p* < 0.05, ***p* < 0.01, ****p* < 0.001, ns = not significant; *p* values were determined using one way ANOVA in E, two-way ANOVA in F.


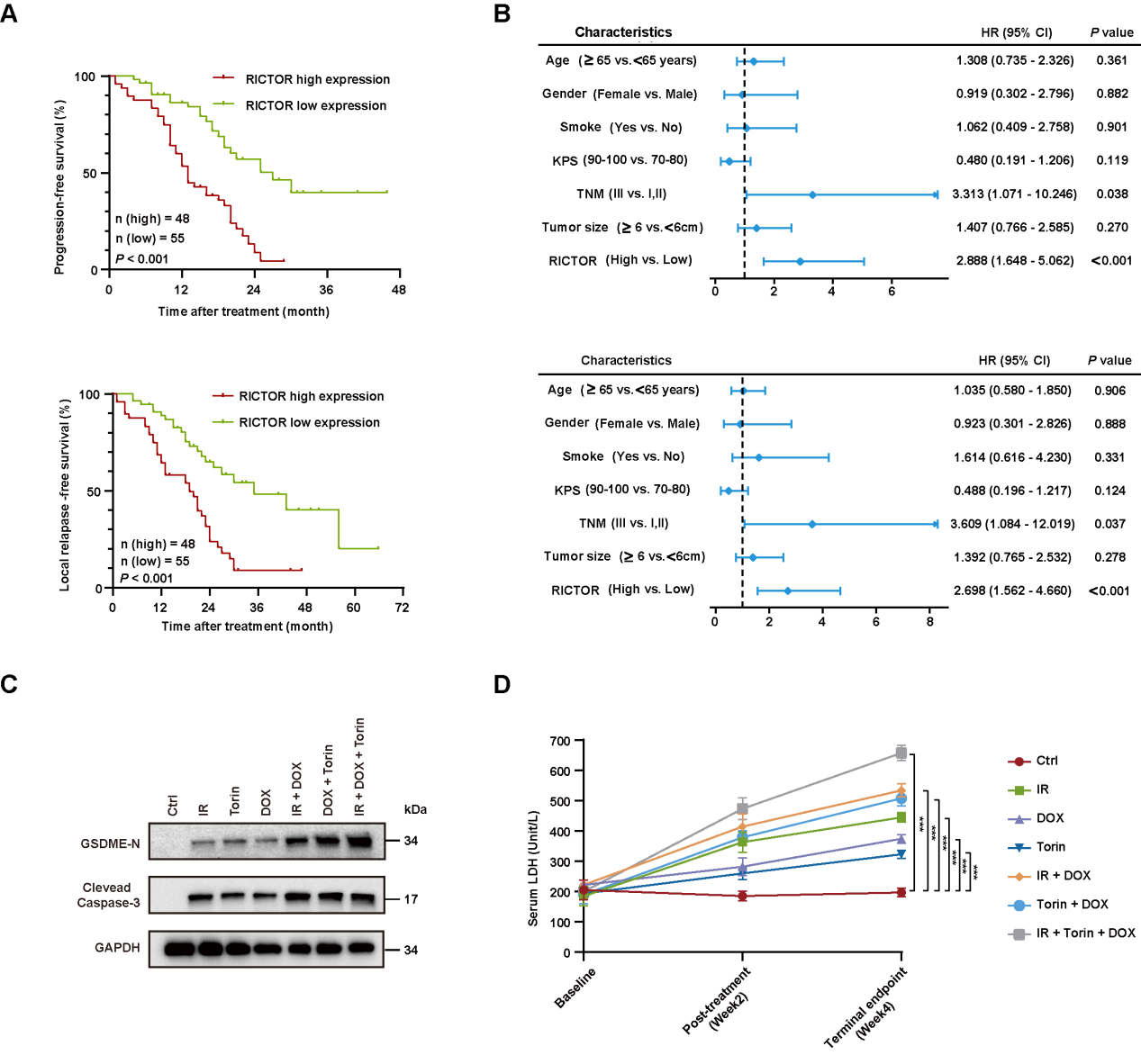


**Supplementary Figure S7. Hyperactivated mTORC2 leads to radioresistance in vivo and is associated with poor prognosis in patients with SCLC.** (A) Kaplan-Meier curves for progression-free survival (PFS) and local relapse-free survival (LRFS) stratified by RICTOR expression levels; *P*-values determined by log-rank test. (B) Forest plots from multivariate Cox regression analysis demonstrating the prognostic impact of RICTOR expression on PFS and LRFS in SCLC. (C) Western blotting was performed to detect the protein expression levels of GSDME-N and Cleaved Caspase-3 in the seven experimental groups: Control, IR, DOX, TORIN, IR+TORIN, DOX+TORIN, and IR+DOX+TORIN. (D) The release of LDH was detected in the seven experimental groups: Control, IR, DOX, TORIN, IR+TORIN, DOX+TORIN, and IR+DOX+TORIN (n = 5). Data are representative of three independent experiments in C and D. Results are presented as mean ± SD; **p* < 0.05, ***p* < 0.01, ****p* < 0.001, ns = not significant; *p* values were determined using two-way ANOVA.

**Supplementary Tables**

**Supplementary Table S1. List of primers, sgRNA and siRNA sequences used in this study.**

| **Gene** | **Sequence** |
| --- | --- |
|  |  |
| **siRNA sequences** |  |
| siRNA-CCT5#1 | GGAUGGAGAUGUGACUGUAAC |
| siRNA-CCT5#2 | GGAUGUGACCUCUGUCGAAGA |
| siRNA-CCT5#3 | GCAACAGCAUGUCAUAGAAAC |
| siRNA-UBA52#1 | CAAGAAGAAGGUCAAAUAA |
| siRNA-UBA52#2 | GCACUCUCUCAGACUACAACA |
| siRNA-UBA52#3 | CCAAGAAGAAGGUCAAAUAA |
| siRNA-H2AC20#1 | GCAACGACGAGGAACUGAACA |
| siRNA-H2AC20#2 | AGGCCGUUCUGUUACCAAAGA |
| siRNA-H2AC20#3 | GCUUGCUGCGCAAAGGCAACU |
| siRNA-H2AC1#1 | GCAAUGAUGAGGAACUCAAUA |
| siRNA-H2AC1#2 | GGCAAUGCGUCUCGCGAUAAC |
| siRNA-H2AC1#3 | GCAGUGUUAGAGUAUCUCACA |
| siRNA-WDR82#1 | ACAAAUACAUCAGAUACUUTT |
| siRNA-WDR82#2 | GCGGUAUAAAAGUAGCUGUTT |
| siRNA-WDR82#3 | GGUGAAGUCAUCAGUUCCGTT |
| siRNA-GRWD1#1 | UCUGUAAGACCACUCCCACTT |
| siRNA-GRWD1#2 | GGAGGACCUGCAGUGGUCATT |
| siRNA-GRWD1#3 | CUGGGACCUUCGGCAGUUCTT |
| siRNA-RBBP7#1 | CAUCAAUGAAGAAUAUAAATT |
| siRNA-RBBP7#2 | UGUGGCCUGGCACCUGCUGTT |
| siRNA-RBBP7#3 | ACGAGAAAUGUUUCUGUUGTT |
| siRNA-RBBP4#1 | GUUUUGGUUCAGUUAGUGGTT |
| siRNA-RBBP4#2 | UGAUGAUCAGAAACUUAUGTT |
| siRNA-RBBP4#3 | GCAAGUGUGGCAAAUGGCATT |
| siRNA-WDR12#1 | UACACUGAUAACAAGAAAUTT |
| siRNA-WDR12#2 | GCUAAAGAUCUGGUCUACATT |
| siRNA-WDR12#3 | UGAAAGUGAACAAUAAUUUTT |
| siRNA-DDB1#1 | CACCUACUAUUUGCUUUGUTT |
| siRNA-DDB1#2 | AAAGCUCUGGUCAGUGAAUTT |
| siRNA-DDB1#3 | CAAGGGCAGGGGGCCCCUUTT |
| siRNA-DDB2#1 | AGGGCUCCGGUCCUAGCAGTT |
| siRNA-DDB2#2 | CGUGGGGAACGUGAUCCUGTT |
| siRNA-DDB2#3 | AGGGCCAAAAGUAUCCAAGTT |
| siRNA-RICTOR-Homo-3032 | GCCAGUGGUUCCAGAUGAUTT |
| siRNA-RICTOR-Homo-763 | GCGAGCUGAUGUAGAAUUATT |
| siRNA-RICTOR-Homo-1105 | CCAAGACAGUUGGAGGCUUTT |
| siRNA-RICTOR-Homo-4250 | GCCUUAAGUUAUGCAUCAUTT |
| siRNA-CUL4B-homo-769 | GCCACGUACCGAUACAGAATT |
| siRNA-CUL4B-homo-1424 | GGAGUUAUUUAGGGCUCAUTT |
| siRNA-CUL4B-homo-2991 | GCUGCAAUUGUUCGAAUUATT |
| siRNA-CUL4B-homo-1785 | GGUGAACACUUAACAGCAATT |
| siRNA-GSDME#1 | GCTTCTAAGTCTGGTGACAAA |
| siRNA-GSDME#2 | GATGATGGAGTATCTGATCTT |
| siRNA-GSDME#3 | GCATGATGAATGACCTGACTT |
| siRNA-GSDME#4 | GCGGTCCTATTTGATGATGAA |
| siRNA-GSDME#5 | GCATTCATAGACATGCCAGAT |
|  |  |
| **sgRNA sequences** |  |
| sgNC | TCCCCTCCACCCCACAGTG |
| sgGSDME | AGTCTTCATTTGGAACCCTG |
| sgCul4B | GCAGTGGATCGAATATATCA |
|  |  |
| **shRNA sequence** |  |
| shGSDME | GATGATGGAGTATCTGATCTT |
|  |  |
| **Real time RT-PCR** |  |
| **primers** |  |
| WDR82-qPCR-F | 5'-TGCTTCGATTTCAGCCCCAA-3' |
| WDR82-qPCR-R | 5'-GGGTTCTCTTTGGTTTGCCC-3' |
| GRWD1-qPCR-F | 5'-GGCCTATGTGCTCTACCACC-3' |
| GRWD1-qPCR-R | 5'-GGGTCCCAGCACACAAGTAA-3' |
| RBBP7-qPCR-F | 5'-CACGTGCATTTGTCTTCCCG-3' |
| RBBP7-qPCR-R | 5'-CCCCAGCACTAGCCAATGAA-3' |
| RBBP4-qPCR-F | 5'-CCGACAAGGAAGCCTTCGAC-3' |
| RBBP4-qPCR-R | 5'-TCACTGTCGTAGTGTGACGC-3' |
| WDR12-qPCR-F | 5'-GTTTCTGCGAATGCCCTTGG-3' |
| WDR12-qPCR-R | 5'-TTCACCCAGGCCACATCTTT-3' |
| DDB1-qPCR-F | 5'-TGGGCACAGCAATGGTGTAT-3' |
| DDB1-qPCR-R | 5'-GCACCGTGCTATTGATGCTG-3' |
| DDB2-qPCR-F | 5'-CCCTGAACCCATGCTGTGAT-3' |
| DDB2-qPCR-R | 5'-GATCTCGCTCTTCTGGTCCG-3' |
| GSDME-qPCR-F1 | 5'-CGGGCGCGCGGATAAT-3' |
| GSDME-qPCR-R1 | 5'-CGACCACTGGACTCGGAAAT-3' |
| PIK3CA-qPCR-F1 | 5'-CAATCGGTGACTGTGTGGGA-3' |
| PIK3CA-qPCR-R1 | 5'-ACAGGTCAATGGCTGCATCA-3' |
| AKT1-qPCR-F1 | 5'-GGACAAGGACGGGCACATTA-3' |
| AKT1-qPCR-R1 | 5'-CGACCGCACATCATCTCGTA-3' |
| mTOR-qPCR-F1 | 5'-TCGCTGAAGTCACACAGACC-3' |
| mTOR-qPCR-R1 | 5'-CTTTGGCATATGCTCGGCAC-3' |
| 4EBP1-qPCR-F1 | 5'-CAAGGGATCTGCCCACCATT-3' |
| 4EBP1-qPCR-R1 | 5'-AACTGTGACTCTTCACCGCC-3' |
| p70-S6K-qPCR-F1 | 5'-GGGGCTATGGAAAGGCAATGA-3' |
| p70-S6K-qPCR-R1 | 5'-CTGCCAAGTAAAAGCAGGCAG-3' |
| P85-qPCR-F1 | 5'-AAGTGCCAGAGTGAAGTGGC-3' |
| P85-qPCR-R1 | 5'-GTCCCGTCTGCTGTATCTCG-3' |

**Supplementary Table S2. List of antibodies used in this study.**

| **Antibody** | **Company** | **Catalog no.** | **Dilution** |
| --- | --- | --- | --- |
| **Western blot** |  |  |  |
| MYC tag Monoclonal antibody | Proteintech | 60003-2-IG | 1:2000 |
| DFNA5/GSDME Monoclonal antibody | Abcam | 215191 | 1:1000 |
| PI3 Kinase p85 (19H8) Rabbit Monoclonal Antibody | Cell signaling technology | # 4257 | 1:1000 |
| Phospho-PI3 Kinase p85 (Tyr458)/p55 (Tyr199) Antibody | Cell signaling technology | #4228 | 1:1000 |
| Cleaved Caspase-3 (Asp175) Rabbit mAb | Cell signaling technology | #9661 | 1:1000 |
| Phospho-mTOR (Ser2448) Monoclonal antibody | Proteintech | 67778-1-Ig | 1:1000 |
| AKT Polyclonal antibody | Proteintech | 10176-2-AP | 1:2000 |
| Phospho-AKT (Ser473) | Proteintech | 80455-1-RR | 1:2000 |
| DYKDDDDK tag Monoclonal antibody (Binds to FLAG® tag epitope) | Proteintech | 66008-4-Ig | 1:5000 |
| DYKDDDDK tag Recombinant antibody (Binds to FLAG® tag epitope) | Proteintech | 80010-1-RR | 1:1000 |
| Cas9 Polyclonal antibody | Proteintech | 26758-1-AP | 1:1000 |
| HRP-conjugated Affinipure Goat Anti-Rabbit IgG(H+L) | Proteintech | SA00001-2 | 1:2000 |
| CUL4B Polyclonal antibody | Proteintech | 12916-1-AP | 1:1000 |
| GFP tag Polyclonal antibody | Proteintech | 50430-2-AP | 1:1000 |
| ubiquitin Polyclonal antibody | Proteintech | 10201-2-AP | 1:1000 |
| V5-tag Polyclonal antibody | Proteintech | 14440-1-AP | 1:2000 |
| ATP1A1 Polyclonal antibody | Proteintech | 14418-1-AP | 1:5000 |
| ERK1/2 Polyclonal antibody | Proteintech | 11257-1-AP | 1:2000 |
| Histone-H3 Polyclonal antibody | Proteintech | 17168-1-AP | 1:2000 |
| 4EBP1 Monoclonal antibody | Proteintech | 60246-1-IG | 1:1000 |
| p70(S6K) Monoclonal antibody | Proteintech | 66638-1-IG | 1:1000 |
| mTOR Monoclonal antibody | Proteintech | 66888-1-IG | 1:5000 |
| HA-Tag (C29F4) Rabbit mAb | Cell signaling technology | #3724 | 1:1000 |
| RICTOR(53A2) Rabbit mAb | Cell signaling technology | #2114 | 1:1000 |
| RAPTOR (24C12) Rabbit mAb | Cell signaling technology | #2280 | 1:1000 |
| Phospho-4E-BP1 (Thr37/46) (236B4) Rabbit mAb (PE Conjugate) | Cell signaling technology | #7547 | 1:1000 |
| Phospho-p70 S6 Kinase (Thr389) (108D2) Rabbit mAb | Cell signaling technology | #9234 | 1:1000 |
|  |  |  |  |
| **Immunofluorescence** |  |  |  |
| DFNA5/GSDME Polyclonal antibody | Abcam | 215191 | 1:1000 |
| Anti-RICTOR antibody | Abcam | ab104838 | 1:500 |
|  |  |  |  |
| **Immunohistochemistry** |  |  |  |
| DFNA5/GSDME Monoclonal antibody | Abcam | 215191 | 1:1000 |
| Anti-RICTOR | Abcam | ab70374 | 1:500 |
|  |  |  |  |
| **Co-Immunoprecipitation** |  |  |  |
| DFNA5/GSDME Polyclonal antibody | Proteintech | 13075-1-AP | 1:1000 |
| DYKDDDDK tag Monoclonal antibody (Binds to FLAG® tag epitope) | Proteintech | 66008-4-Ig | 1:1000 |
| V5-tag Polyclonal antibody | Proteintech | 14440-1-AP | 1:1000 |
| HA-Tag (C29F4) Rabbit mAb | Cell signaling technology | #3724 | 1:50 |
| Myc-Tag (71D10) Rabbit mAb | Cell signaling technology | 2278s | 1:200 |

*Cell signaling Technology (CST, Danvers, Massachusetts, USA); *Abcam (Cambridge, Cambridgeshire, United Kingdom); *Proteintech (Wuhan, Hubei Province, China)

**Supplementary Table S3. Univariate and multivariate logistic regression analysis of prognostic factors in 103 patients with SCLC.**

| **Variable** | **Univariate analysis** | | | **Multivariate analysis** | | |
| --- | --- | --- | --- | --- | --- | --- |
|  | **HR** | **95% CI** | ***P* value** | **HR** | **95% CI** | ***P* value** |
| **RICTOR** |  |  |  |  |  |  |
| Age ($\geq$65 vs. < 65 years) | 0.960 | 0.428 - 2.151 | 0.920 | 0.771 | 0.329 - 1.807 | 0.549 |
| Gender (Female vs. Male) | 2.149 | 0.717 - 6.438 | 0.172 | 3.248 | 0.545 - 19.359 | 0.196 |
| Smoke (Yes vs. No) | 1.333 | 0.527 - 3.377 | 0.544 | 0.709 | 0.156 - 3.217 | 0.656 |
| KPS (90-100 vs. 70-80) | 1.171 | 0.379 - 3.615 | 0.784 | 1.242 | 0.349 - 4.415 | 0.738 |
| TNM Stage (III vs. I, II) | 0.409 | 0.119 - 1.402 | 0.155 | 0.373 | 0.091 - 1.530 | 0.171 |
| Tumor size ($\geq$ 6 vs. < 6 cm) | 0.797 | 0.365 - 1.739 | 0.568 | 0.898 | 0.371 - 2.176 | 0.812 |

*P* value is determined by logistic regression analysis. All patients were restaged according to the 8th edition of the AJCC Cancer Staging Manual.
