# Supplementary material for: mTORC2 Phosphorylation of GSDME‐N Drives Cullin4B‐Mediated Proteasomal Degradation to Suppress Pyroptosis and Confer Radioresistance in Small Cell Lung Cancer
Source: Adv Sci (Weinh). 2026 May 27:e75844. Online ahead of print. doi: 10.1002/advs.75844 (PMC13335795; doi:10.1002/advs.75844)

Fig 2A

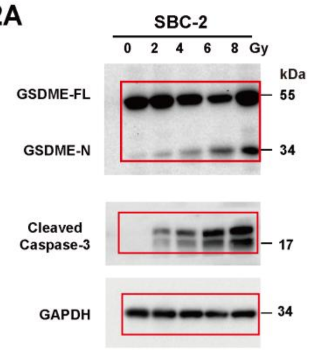

Fig 2C

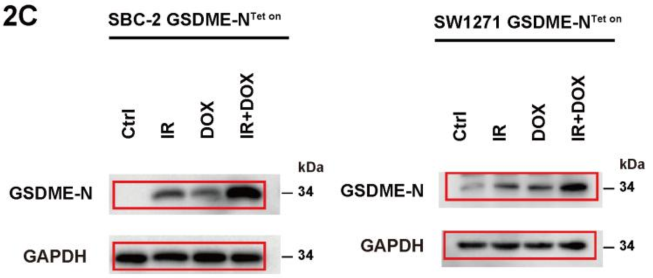

Fig 2G

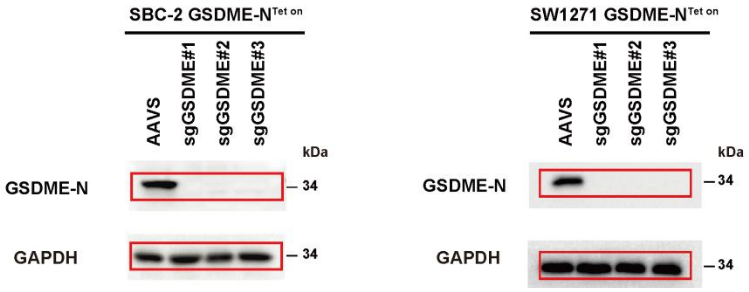

Fig 3E

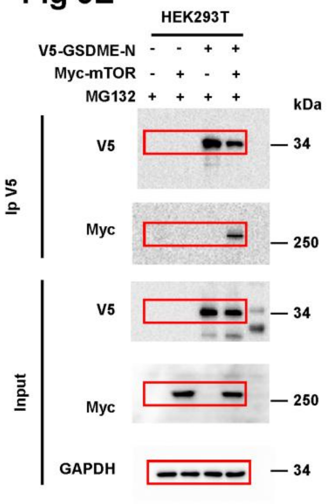

Fig 3F

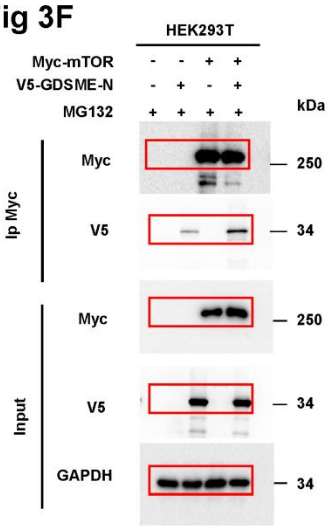

Fig 3G

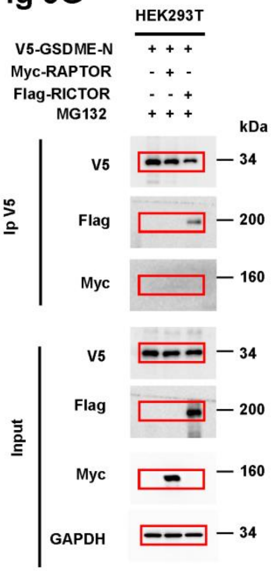

Fig 3H

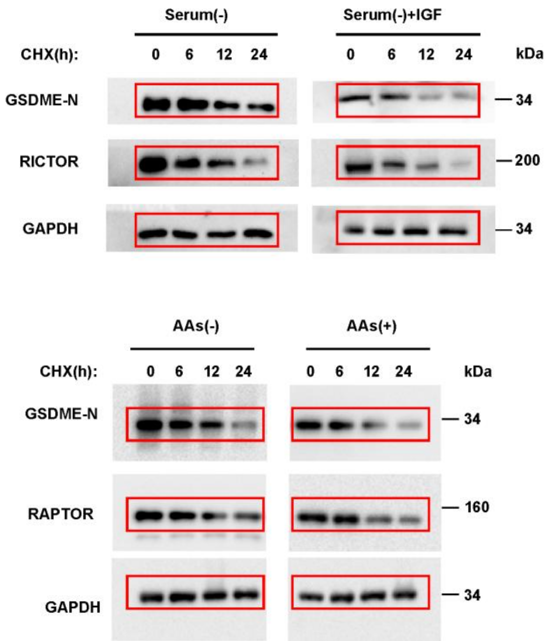

Fig 3I

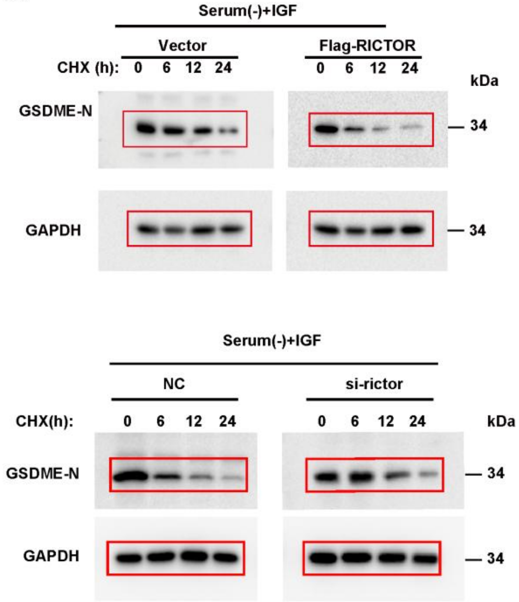

Fig 4A

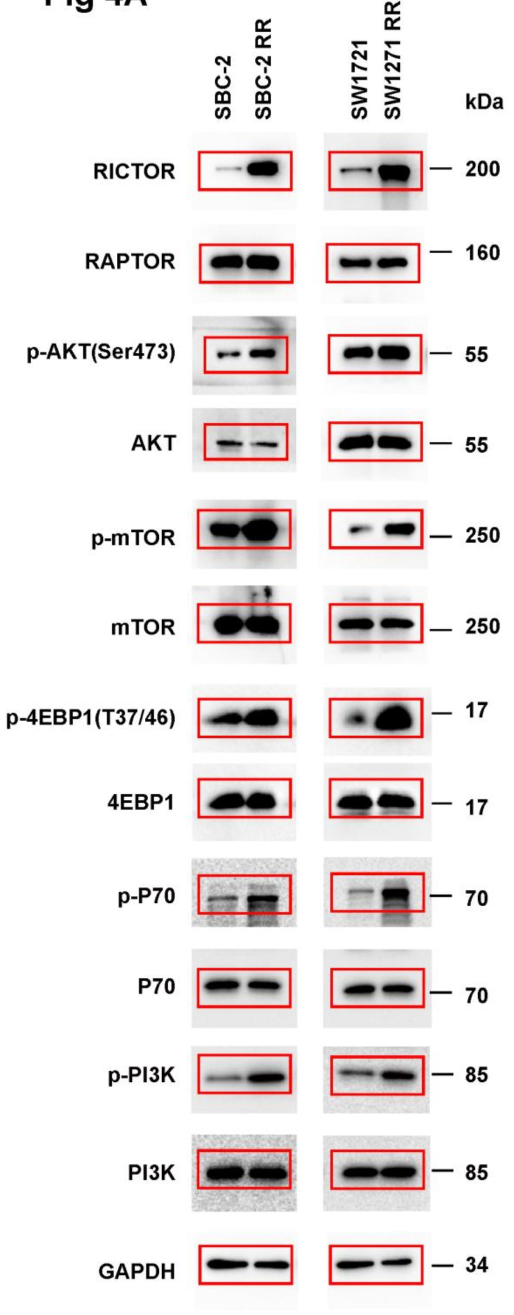
SBC-2
SBC-2 RR
SW1721
SW1271 RR
kDa
RICTOR
200
RAPTOR
160
p-AKT(Ser473)
55
AKT
55
p-mTOR
250
mTOR
250
p-4EBP1(T37/46)
17
4EBP1
17
p-P70
70
P70
70
p-PI3K
85
PI3K
85
GAPDH
34

Fig 4F

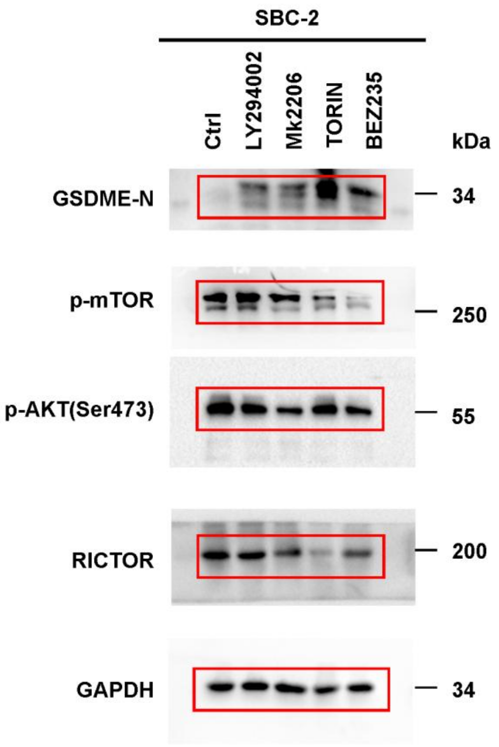
SBC-2
Ctrl
LY294002
Mk2206
TORIN
BEZ235
kDa
GSDME-N
34
p-mTOR
250
p-AKT(Ser473)
55
RICTOR
200
GAPDH
34

Fig 5A

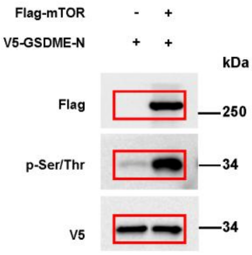

Fig 5B

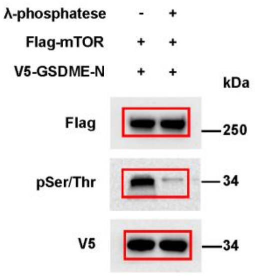

Fig 5D

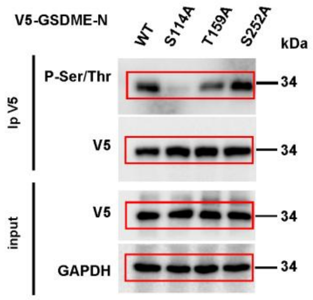

Fig 5F

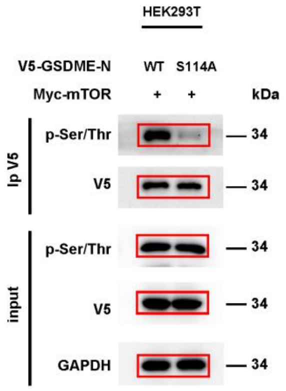

Fig 5E

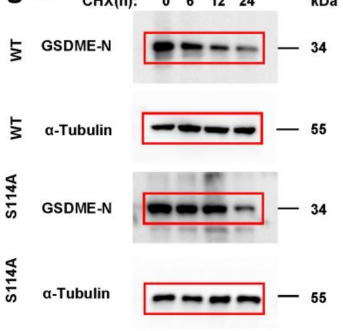

Fig 6A

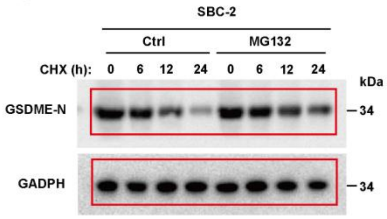

Fig 6D

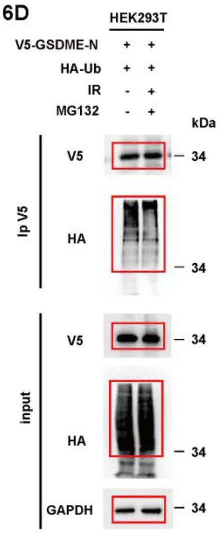

Fig 6E

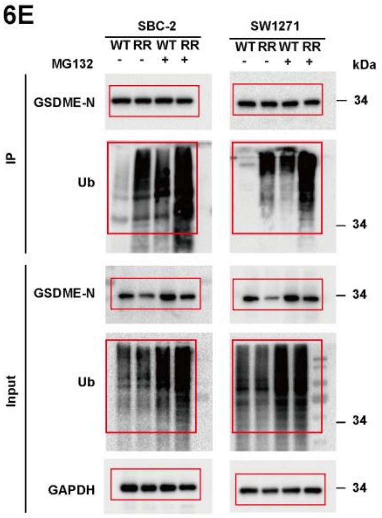

Fig 6F

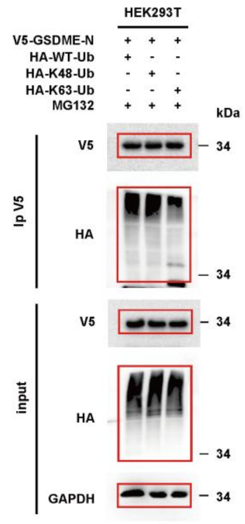

Fig 6G

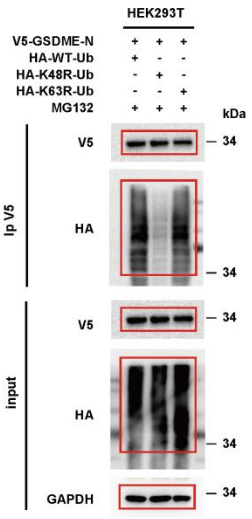

Fig 6I

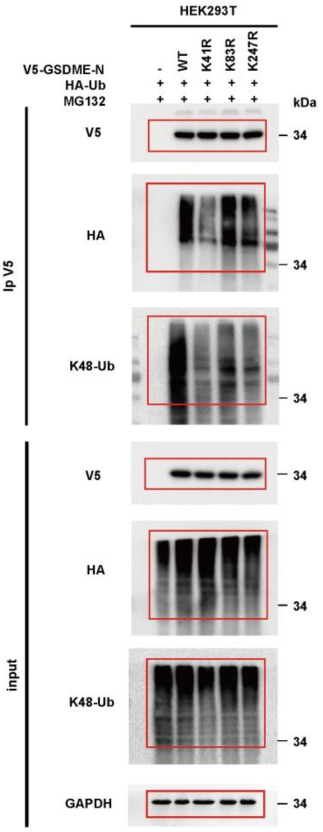

Fig 6J

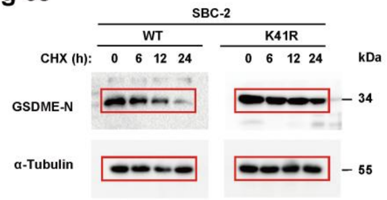

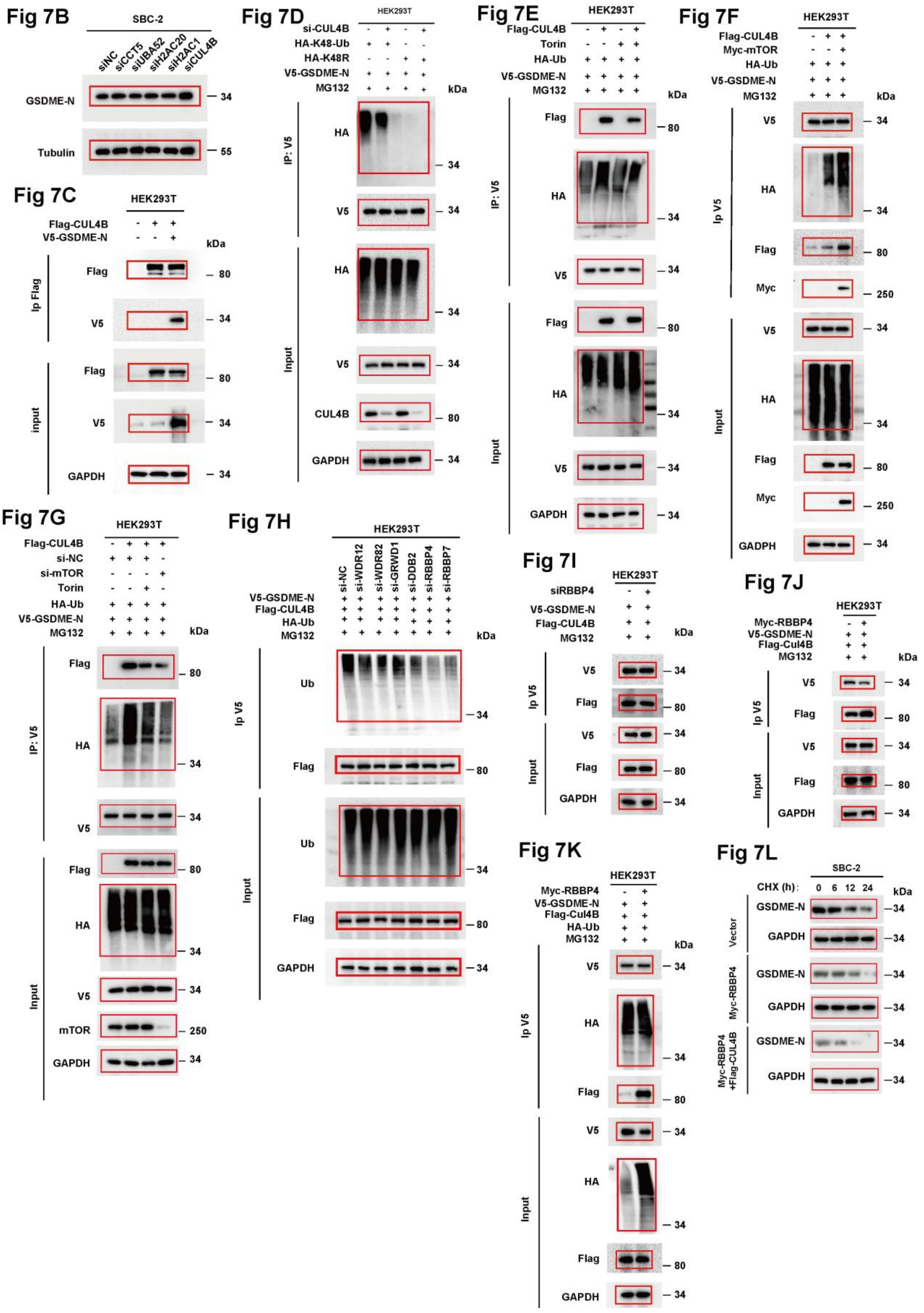
Fig 7B
SBC-2
siNC
siCCT5
siUBA52
siH2AC20
siH2AC1
siCUL4B
GSDME-N
Tubulin
34
55
Fig 7C
HEK293T
Flag-CUL4B
V5-GSDME-N
kDa
Ip Flag
input
Flag
V5
GAPDH
80
34
Fig 7D
HEK293T
si-CUL4B
HA-K48-Ub
HA-K48R
V5-GSDME-N
MG132
kDa
IP: V5
Input
HA
V5
CUL4B
GAPDH
34
80
Fig 7E
HEK293T
Flag-CUL4B
Torin
HA-Ub
V5-GSDME-N
MG132
kDa
IP: V5
Input
Flag
HA
V5
GAPDH
80
34
Fig 7F
HEK293T
Flag-CUL4B
Myc-mTOR
HA-Ub
V5-GSDME-N
MG132
kDa
Ip V5
Input
V5
HA
Flag
Myc
GADPH
34
80
250
Fig 7G
HEK293T
Flag-CUL4B
si-NC
si-mTOR
Torin
HA-Ub
V5-GSDME-N
MG132
kDa
IP: V5
Input
Flag
HA
V5
mTOR
GAPDH
80
34
250
Fig 7H
HEK293T
si-NC
si-WDR12
si-WDR82
si-GRWD1
si-DDB2
si-RBBP4
si-RBBP7
V5-GSDME-N
Flag-CUL4B
HA-Ub
MG132
kDa
Ip V5
Input
Ub
Flag
GAPDH
34
80
Fig 7I
HEK293T
siRBBP4
V5-GSDME-N
Flag-CUL4B
MG132
kDa
Ip V5
Input
V5
Flag
GAPDH
34
80
Fig 7J
HEK293T
Myc-RBBP4
V5-GSDME-N
Flag-Cul4B
MG132
kDa
Ip V5
Input
V5
Flag
GAPDH
34
80
Fig 7K
HEK293T
Myc-RBBP4
V5-GSDME-N
Flag-Cul4B
HA-Ub
MG132
kDa
Ip V5
Input
V5
HA
Flag
GAPDH
34
80
Fig 7L
SBC-2
CHX (h):
0
6
12
24
kDa
Vector
Myc-RBBP4
Myc-RBBP4 +Flag-CUL4B
GSDME-N
GAPDH
34

Fig S2A

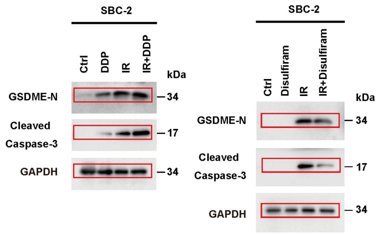

Fig S3B

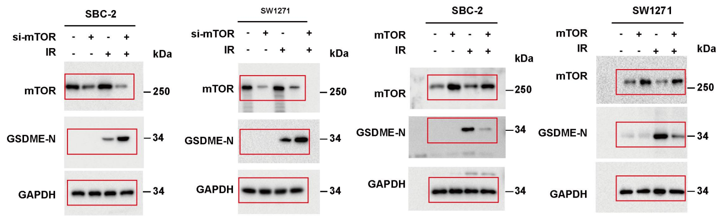

Fig S3H

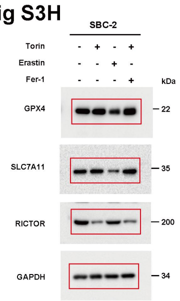

Fig S3I

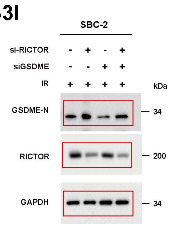

Fig S4G

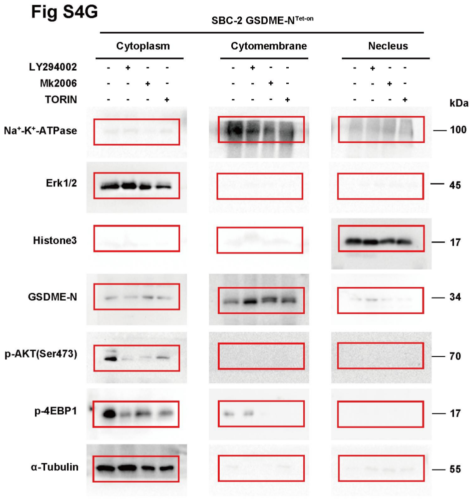

Fig S5A

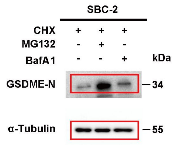

Fig S5B

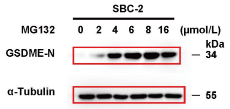

Fig S6B

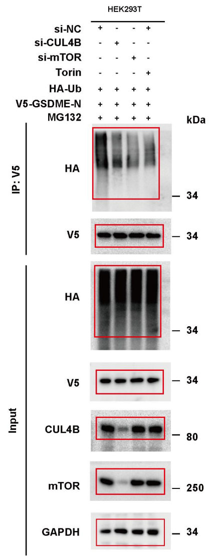

Fig S7C

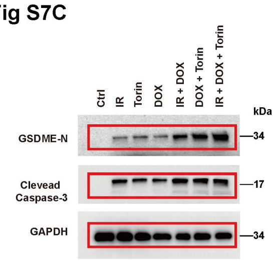

Supplement: Supplementary file 2 — Supporting File 2: advs75844‐sup‐0002‐blotts.zip. [file ADVS-9999-e75844-s001.zip › advs75844-sup-0002-blotts/Western blotting.pdf]
